# Relevance of ATM Status in Driving Sensitivity to DNA Damage Response Inhibitors in Patient-Derived Xenograft Models

**DOI:** 10.3390/cancers15164195

**Published:** 2023-08-21

**Authors:** Ankur Karmokar, Rebecca Sargeant, Adina M. Hughes, Hana Baakza, Zena Wilson, Sara Talbot, Sarah Bloomfield, Elisabetta Leo, Gemma N. Jones, Maria Likhatcheva, Luis Tobalina, Emma Dean, Elaine B. Cadogan, Alan Lau

**Affiliations:** 1 Bioscience, Oncology R&D, AstraZeneca, Cambridge CB2 0AA, UK; 2 Imaging & Data Analytics, Clinical Pharmacology & Safety Sciences, BioPharmaceuticals R&D, AstraZeneca, Cambridge CB2 0AA, UK; 3 Translational Medicine, Oncology R&D, AstraZeneca, Cambridge CB2 0AA, UK; 4 Oncology Data Science, Oncology R&D, AstraZeneca, Cambridge CB2 0AA, UK; 5 Oncology R&D, AstraZeneca, Cambridge CB2 0AA, UK

**Keywords:** DNA damage response, ATM, biallelic mutation, truncating mutation, biomarker, immunohistochemistry

## Abstract

**Simple Summary:**

The inactivation of DNA damage response (DDR) pathways provides new opportunities to target cancers. One of the key DDR kinases is ATM which is reported to be mutated across a wide range of solid and haematological cancers. There is contrasting evidence on how ATM alterations in patients could enhance treatment responses to DDR inhibitors. In this pre-clinical study, we attempted to understand (1) how different types of ATM mutation correlate with protein expression/loss and (2) which ATM alterations could predict sensitivity to different DDR inhibitors both as monotherapy and combination therapy in a range of patient-derived xenograft models.

**Abstract:**

Ataxia-telangiectasia mutated gene (ATM) is a key component of the DNA damage response (DDR) and double-strand break repair pathway. The functional loss of ATM (ATM deficiency) is hypothesised to enhance sensitivity to DDR inhibitors (DDRi). Whole-exome sequencing (WES), immunohistochemistry (IHC), and Western blotting (WB) were used to characterise the baseline ATM status across a panel of ATM mutated patient-derived xenograft (PDX) models from a range of tumour types. Antitumour efficacy was assessed with poly(ADP-ribose)polymerase (PARP, olaparib), ataxia- telangiectasia and rad3-related protein (ATR, AZD6738), and DNA-dependent protein kinase (DNA-PK, AZD7648) inhibitors as a monotherapy or in combination to associate responses with ATM status. Biallelic truncation/frameshift ATM mutations were linked to ATM protein loss while monoallelic or missense mutations, including the clinically relevant recurrent R3008H mutation, did not confer ATM protein loss by IHC. DDRi agents showed a mixed response across the PDX’s but with a general trend toward greater activity, particularly in combination in models with biallelic ATM mutation and protein loss. A PDX with an ATM splice-site mutation, 2127T > C, with a high relative baseline ATM expression and KAP1 phosphorylation responded to all DDRi treatments. These data highlight the heterogeneity and complexity in describing targetable ATM-deficiencies and the fact that current patient selection biomarker methods remain imperfect; although, complete ATM loss was best able to enrich for DDRi sensitivity.

## 1. Introduction

Targeting the DNA damage response (DDR) pathway in cancer has been of great interest in the clinic over the recent years, mainly due to the fact that cancer cells demonstrate greater genomic instability and potential dependency on targetable DNA repair pathways [1]. One such example is the approval of poly(ADP-ribose)polymerase (PARP) inhibitors such as olaparib, rucaparib, niraparib, and talazoparib for the treatment of solid tumours including breast, ovarian, prostate, and pancreatic cancers. These agents are particularly useful in patients with inactivating BReast CAncer gene *BRCA* mutations (*BRCA*m), including *BRCA1* and *BRCA2*, or aberrant homologous recombination repair (HRR) pathway deficiencies [2,3,4,5,6,7,8]. Approved PARP inhibitors primarily act through catalytic inhibition in PARP1/2 and the subsequent stabilisation or “trapping” of PARP proteins onto DNA which causes physical blocks to DNA replication, replication fork stalling/collapse, and increased DNA double-strand breaks (DSBs). In normal cells, DSBs are repaired by the homologous recombination repair (HRR) pathway but in *BRCA* mutant or HRR deficient cells they cannot be faithfully repaired and ultimately lead to cell death, a concept known as “synthetic lethality.” In addition to *BRCA1*/*2*, studies have identified other candidate genes that confer sensitivity to PARP inhibition such as the ataxia-telangiectasia mutated gene (*ATM*) [9,10,11,12].

ATM belongs to the PI3K-like family of serine–threonine protein kinases and forms an integral part of the DDR response pathway machinery, namely responding to DNA DSBs and coordinating the G1/S cell cycle checkpoint [12,13,14]. Analysis of *ATM* mutation frequency in The Cancer Genome Atlas cohort indicated that *ATM* is mutated in approximately 5% of all cancers [15,16]. The mutation frequency of *ATM* is highest in mantle cell lymphoma at 40% followed by colorectal cancer at 20% and prostate and lung cancer at 10%. Cell lines derived from patients with ataxia-telangiectasia and from engineered *ATM* knockout human or mouse models are sensitive to DNA-damaging agents [12] and PARP inhibitors, raising the possibility that cancers with a functional loss of ATM could be particularly sensitive to these agents [17,18,19,20]. More recently, inhibition of the DDR signalling kinases ataxia-telangiectasia and rad3-related protein (ATR) and DNA-dependent protein kinase (DNA-PK) has also been linked to enhanced activity in pre-clinical models of functional ATM loss. Although ATM, ATR, and DNA-PK are related to DDR PI3K-like protein kinases, they normally respond to distinct types of DNA damage in different phases of the cell cycle [13]. ATR is activated by and facilitates the repair of stalled DNA replication forks (also known as replication stress) and coordinates the S/G2 cell cycle checkpoints [21,22,23]. DNA-PK is mainly involved in coordinating the repair of frank double-ended DSBs by the non-homologous end joining pathway [1]. Given the interplay between DNA repair pathways [1,13], the loss of function of ATM in cancer cells could create an enhanced reliance on the remaining ATR or DNA-PK dependent pathways to repair DNA damage. Consistent with this hypothesis, studies have demonstrated enhanced activity of PARP, ATR, and DNA-PK inhibitors in pre-clinical models with ATM loss of function, characterised by increased apoptosis, DNA damage, chromosomal breaks, and micronuclei formation along with G2/M cell cycle arrest (PARP and DNA-PK) or abrogation of the G2/M cell cycle checkpoint (ATR) when compared to ATM proficient models. *ATM* knockout models of pancreatic, prostate, and lung cancers have been shown to lead to enhanced efficacy with PARP and/or ATR inhibition [24,25,26]. Similarly, a DNA-PK inhibitor in combination with olaparib showed enhanced activity in *ATM* knockout cells in vitro and in vivo [27].

**Figure A1:**
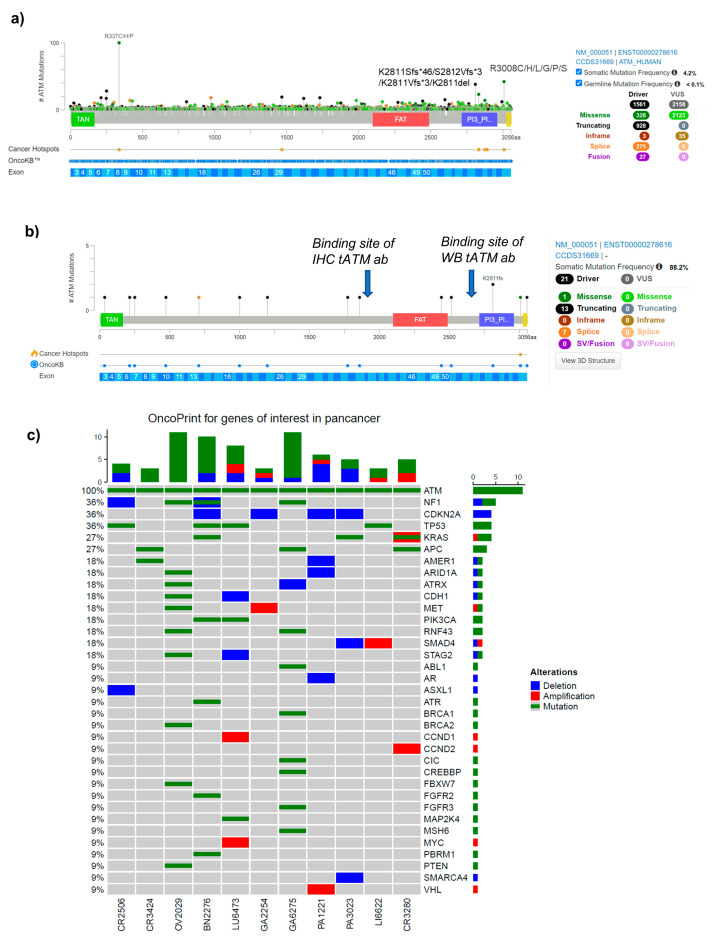
ATM alterations in PDX models. (**a**,**b**) Represents the ATM mutation lollipop in clinical samples and PDX models, respectively. # refers the number of muations, * indicates a truncating ATM alteration, fs indicates a frameshift mutation and del a suspected deletion (**c**) represents the oncoprint of pan-cancer gene alteration across PDX models used in this study.

Data generated in pre-clinical studies have mostly been derived from isogenic cell lines or well-characterised xenografts in which *ATM* has been completely knocked out [28,29,30,31,32]. However, in cancer patients, the functional relevance of most tumour *ATM* alterations is unknown. *ATM* is a large gene and analysis shows hundreds of mutations including missense mutations and truncations, both monoallelic and biallelic, scattered throughout the coding region (Appendix A Figure A1a), with R3008C/H and R337H/C missense mutations as low-frequency “hotspot” mutations [12,14]. It is therefore challenging to interpret mutation profiles of *ATM* with respect to their loss of function for patient selection. Indeed, although some studies have considered the loss of ATM protein expression by immunohistochemistry (IHC) as a surrogate for loss of function or deleterious *ATM* mutations, protein expression may not solely constitute a functional endpoint (e.g., “dominant negative” expression) and may be hampered by limits of detection. Therefore, in addition to protein expression, functional assays along with assessments of downstream targets of ATM should be considered [23]. Furthermore, in the clinic, it remains unclear whether patients with tumours harbouring *ATM* alterations would benefit from DDR inhibitor treatments, particularly as monotherapy, and importantly, there is no generally accepted best strategy to define ATM deficiency for patient selection.

For these reasons, in this study, we aimed to understand how *ATM* mutations correlate with loss of ATM protein. We first selected a panel of patient-derived xenograft (PDX) models based on available *ATM* mutation data and characterised them for ATM protein expression by IHC and Western blot. We then assessed the baseline expression of phosphorylated ATM(Ser-1981)(pATM), phosphorylated RAD50(Ser-635)(pRAD50), and phosphorylated KAP1(Ser-824)(pKAP1) as functional markers (direct substrates) of ATM kinase activity as well as γH2AX (phosphorylated Ser-139) as a surrogate marker of active genomic instability (DNA breaks). Finally, we correlated ATM status with the antitumour efficacy of DDR inhibitor (DDRi) agents (ATRi, DNA-PKi, and PARPi) both as monotherapies and in combination with each other. To our knowledge, this is the first dataset of head-to-head comparisons of combination therapies of DDRi agents in a defined series of ATM-altered PDX models.

## 2. Materials and Methods

### 2.1. In Vivo Studies

The in vivo studies were run at Crown Bioscience (San Diego, CA, USA). All in vivo animal experiments were conducted in a facility accredited by the Association for Assessment and Accreditation of Laboratory Animal Care under the guidelines of AstraZeneca’s Institutional Animal Care and Use Committee and appropriate animal research approvals. All procedures involving the use and care of animals were approved by the Institutional Animal Care and Use Committee at Crown Bioscience. In addition, all protocols pertaining to animal usage were approved internally at AstraZeneca via Partner, a centralised database for the ethical assessment and risk management of third-party institutes that conduct animal research for or on behalf of AstraZeneca.

### 2.2. Tumour Inoculation

Fresh tumours from donor animals were harvested and cut into pieces approximately 2–3 mm in diameter. One fragment was subcutaneously implanted into the right dorsal flank of female Balb/c nude mice for tumour development. Once tumour size reached 150–200 mm^3^ and mice were randomised into the study at *n* = 5 per treatment arm. The randomisation was performed using the matched distribution method. The day of randomisation was denoted as day 0.

### 2.3. Experimental Design

Eleven PDX models were used for this study. The treatment arms consisted of monotherapies with AZD6738 at 25 mg/kg and 12.5 mg/kg twice daily (BID), AZD7648 at 100 mg/kg once daily (QD), and olaparib at 100 mg/kg QD or BID. In combination doublets, AZD6738 was combined with olaparib or AZD7648 and AZD7648 was combined with olaparib. Details on the doses and schedules are shown in Table 1.

### 2.4. In Vivo Data Analysis

**Figure A2:**
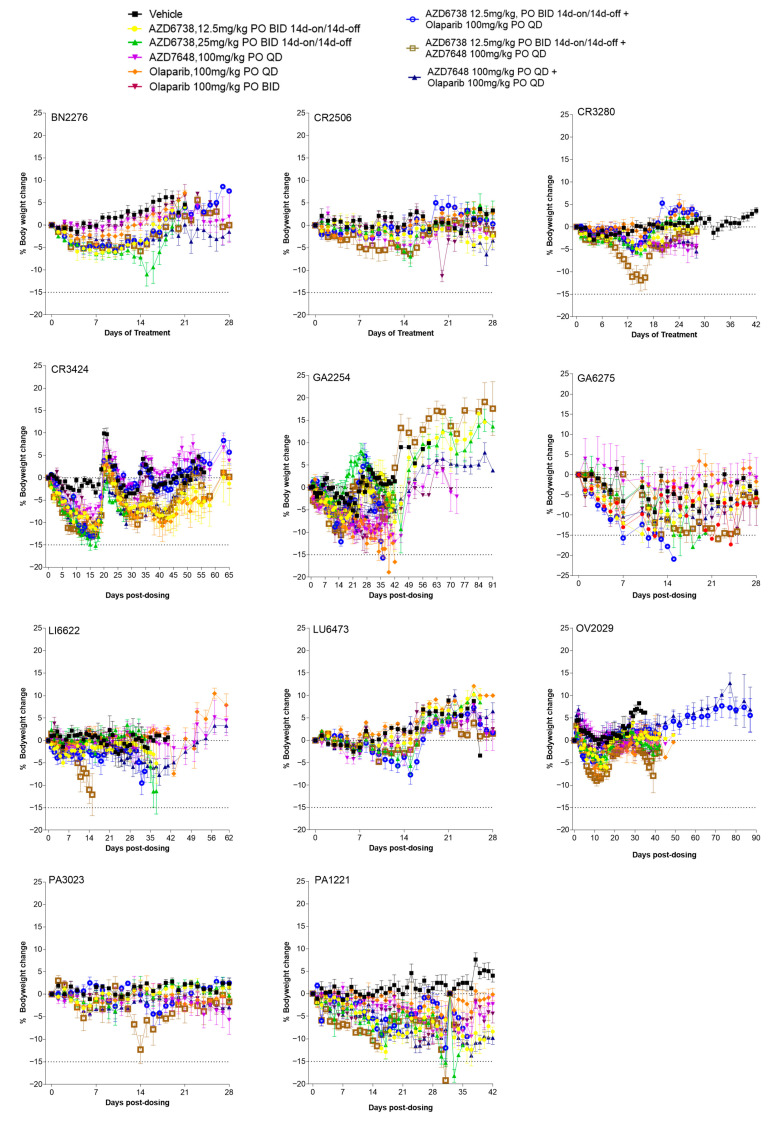
Body weight change following treatment with DDRi agents in 11 PDX models. Body weight change was plotted as the % change in body weight with respect to body weight at the start of dosing. For some models, body weight change beyond the dosing period is also plotted. Values represent the mean ± SEM or individual animal depending on the number of animals remaining in the study.

Tumour growth curves were plotted as geometric relative means of the tumour volume after dosing. Tumour growth inhibition/regression was used as a measure of efficacy in these models. Tumour growth inhibition (TGI) was calculated as the percent change in tumour volume after treatment relative to the change in tumour volume in control animals at a particular time point. Regression was calculated as a percent decrease in tumour volume after treatment relative to tumour volume at the start of treatment. In addition, the best response was used to compare responses across all models and was calculated as the maximum percent tumour growth inhibition after drug treatment. A minimum of 14 days of tumour growth was allowed for each model to measure the best response. Body weight changes were plotted as the percent change in body weight after drug treatment relative to body weight at the start of treatment (Appendix A, Figure A2).

### 2.5. ATM Assessment

IHC and Western blots were performed for baseline assessment of ATM protein in the PDX models. To this end, tumours from vehicle-treated animals were collected once they reached the study endpoint. Tumours were divided into two parts: (1) a snap-frozen sample for Western blot and (2) a formalin-fixed and paraffin-embedded sample for IHC analysis. 

### 2.6. Histology and IHC

The formalin-fixed and paraffin-embedded blocks were sectioned at 3 μM. Haematoxylin–eosin staining was performed on all sections using Mayer’s haematoxylin by a standard automated protocol. Standard automated IHC protocols were used for primary antibody detection of total ATM at a dilution of 1:50 (ab32420; Abcam, Cambridge, UK) and pRAD50 (Ser-635) at a dilution of 1:100 (14223; Cell Signalling Technologies, Danvers, MA, USA) for all samples. 3,3′-Diaminobenzidine horseradish peroxidase-activated chromogen (760-159; Roche Diagnostics, Basel, Switzerland) was used to visualise positive staining in single-plex staining for both ATM and pRAD50 with a haematoxylin counterstain. Haematoxylin–eosin and IHC sections were imaged with an Aperio AT2 Scanscope Console (version 102.0.7.5; Leica, Wetzlar, Germany).

Histological evaluation and quantitative analysis of ATM- and pRAD50-positive cells were conducted by a senior imaging scientist and IHC images were analysed and quantified by using H score analysis algorithms performed on all IHC tissue sections with HALO artificial-intelligence image analysis software (version 3.32541.323; Indica Labs, Albuquerque, NM, USA). Data visualisation was carried out with Prism (version 9.0.2; GraphPad Software, La Jolla, CA, USA).

### 2.7. Tumour Protein Isolation and Immunoblotting

Flash-frozen pieces of tumour were lysed in ice-cold buffer containing tris(hydroxymethyl)aminomethane (Tris)–NaCl, pH 7.5, 20 mmol/L; NaCl, 137 mmol/L; NP40 1%; and 10% glycerol. The buffer was supplemented with NaF, 50 mmol/L; Na_3_VO_4_, 1 mmol/L; Protease Complete Inhibitor Tablet (1836145; Roche); and Phosphatase Inhibitor Cocktails 2 and 3 (P0044 and P5726; Sigma Chemical Co., St. Louis, MO, USA). Homogenization was performed three times using FastPrep tubes (6910-500; MP Biomedicals, Irvine, CA, USA) and FastPrep-24 (MP Biomedicals). All samples were sonicated for 30 s at a high amplitude (Diagenode, Denville, NJ, USA) and then centrifuged at 13,000 rpm at 4 °C for 10 min and the supernatants were collected. The protein concentration was calculated by using Protein Assay Reagent A (23228; Pierce Chemical, Dallas, TX, USA) plus BCA Protein Assay Reagent B (23224; Pierce Chemical). A total of 40 μg of protein was separated on 4–12% sodium dodecyl sulphate–polyacrylamide gel electrophoresis gels (NP0323BOX; Invitrogen, Carlsbad, CA, USA) at 180 V in 1× NuPAGE MES SDS running buffer (NP0002; Invitrogen) in the presence of NuPAGE Antioxidant (NP0005; Invitrogen). Large proteins were separated on 3–8% Tris acetate gels (WG1603BOX; Invitrogen) at 150 V in 1× NuPAGE Tris acetate running buffer (LA0041; Invitrogen) in the presence of NuPAGE antioxidant (NP0005; Invitrogen).

Proteins were electro-transferred to 0.2-μm nitrocellulose membranes (IB3010-01; Invitrogen) using an Iblot dry blotting system (IB1000; Invitrogen). Membranes were blocked for 1 h in 5% milk in Tris-buffered saline–Tween and then hybridized with the primary antibodies in 5% milk in Tris-buffered saline–Tween at 1:1000 dilution with total ATM (tATM) (Abcam ab78), pATM (S1981, Abcam Ab81292), pKAP1 (S824, Abcam Ab81292), and γH2AX (S139, CST2577). Mouse and rabbit horseradish peroxidase-conjugated secondary antibodies (CST #7074 and #7076) were diluted in 5% milk in Tris-buffered saline–Tween and proteins were detected with SuperSignal West Dura Chemiluminescent Substrate reagent (Pierce–Thermo Scientific, Waltham, MA, USA). Biomarker signals were quantified with Genetools software (Syngene, Bangalore, India) on unsaturated images and normalized to vinculin control. 

### 2.8. RAD51 Foci Staining

RAD51/Geminin immunofluorescence (IF) was performed as previously described [33] with small modifications to the staining protocol. Briefly, semi-automated Labvision (Thermo scientific) auto-stainer was used for antibody incubation and the blocking and washing steps. The epitope retrieval step was performed using DAKO target retrieval solution (pH9) in a Whirlpool microwave. Anti-Rad51 (ab133534) and anti-Geminin (NCL-L-Geminin, Leica) antibodies were used at 1/1000 and 1/20, respectively, in blocking buffer (1% BSA DAKO wash buffer, DAKO, S300685). Donkey anti-rabbit Alexa Fluor 568 (A-10042, Invitrogen) and goat anti-mouse Alexa Fluor 488 (A-11001, Invitrogen) were used as secondary antibodies at a concentration of 1/500 in blocking buffer. Hoechst (BD Biosciences, 33342) was used as the nuclear staining at a concentration of 1/1250 in TBS. RAD51 scoring was conducted manually. To confirm RAD51 low scores (<10% RAD51 positive cells), the presence of DNA damage was assessed by γH2AX IF. γH2AX IF staining was conducted using the Leica—BOND RX fully automated platform. Briefly, samples were stained with anti-yH2AX (9718, CST) [2.68 ug/mL] and anti-Geminin [0.98 ug/mL]. Opal 520 [1/400] (FP1487001KT, Akoya) and anti-rabbit Alexa Fluor 568 (A-10042, Invitrogen) were used as secondary antibodies. Stained samples were imaged using AxioImager Z2 (40×) + Metasystems and analysed by imaging analysis using Visiopharm software (3.14.4). Cells were scored as positive for γH2AX when 2 or more foci were detected. According to internally available data obtained from ovarian and breast PDXs, a 10% cut off was used to determine γH2AX-low samples. A sample that presented a low RAD51 score and low yH2AX score was considered not evaluable due to low levels of DNA damage.

### 2.9. Whole-Exome Sequencing (WES)

DNA was extracted with the AllPrep DNA/RNA Micro Kit (Qiagen, Hilden, Germany) and eluted in Tris–ethylenediaminetetraacetic acid buffer. The DNA concentration was determined by a Qubit (Invitrogen), the purity was determined by a NanoDrop 8000 (Thermo Fisher Scientific, Waltham, MA, USA), and DNA integrity was measured with a 4200 TapeStation (Agilent, Santa Clara, CA, USA). All samples had a DNA integrity number of ≥7.0. Libraries were prepared by using the NEBNext Ultra II DNA Library Prep Kit for Illumina (New England Biolabs, Ipswich, MA, USA) and the SureSelect Human All Exon V8 (Agilent). Libraries were subsequently quantified by using the Qubit and KAPA library quantification kit, ROX low (Roche). Library sizes were also determined by a TapeStation 4200 (Agilent). Paired-end sequencing was performed on a NovaSeq 6000 (Illumina, San Diego, CA, USA). Reads were aligned to hg38 using bwa-0.7.17 with >60 million raw reads per sample.

## 3. Results

### 3.1. PDX Model Selection and Baseline Characterisation

**Figure 1:**
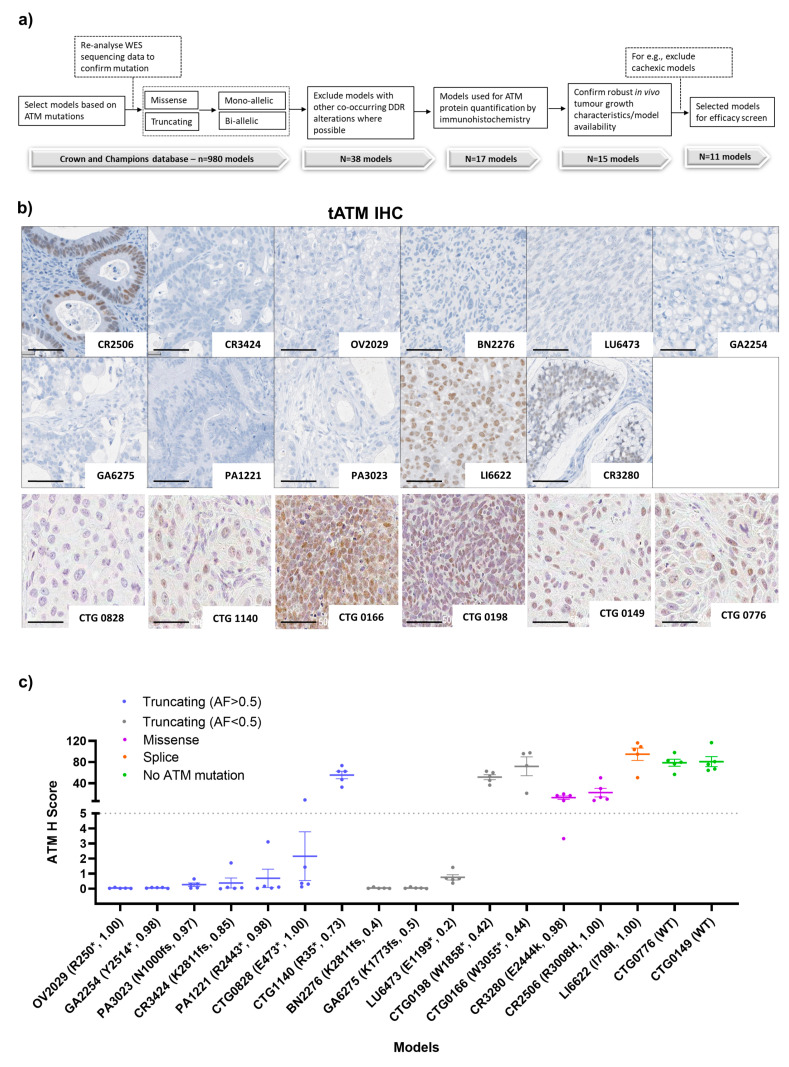
PDX model selection and the baseline profile of ATM protein expression and genetic alterations. (**a**) Strategy for the selection of PDX models, (**b**) representative IHC images of tATM in 17 PDX (scale bar represents 50 µm), and (**c**) comparison between the ATM H score and different type of ATM alterations across 17 PDX models. Letters and numbers in brackets on the *x*-axis denote the ATM protein change and variant allele frequency, respectively. * Indicates the predicted truncating ATM mutation and fs indicates a frameshift mutation. Data represented as the mean ± SEM (*n* = 4 or 5 per model). Images were captured at 40× magnification. For detailed IHC staining, refer to the method section.

**Figure A3:**
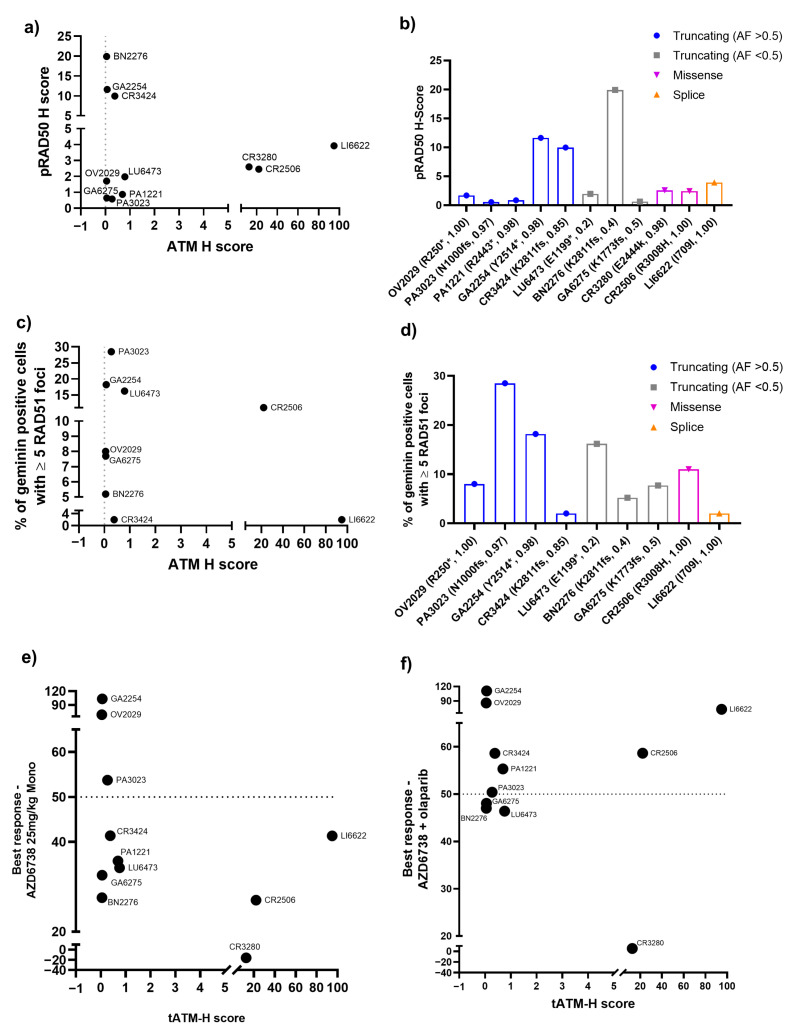
Correlation of ATM expression and mutation types with pRAD50 expression and RAD51 foci. (**a**) Comparison of ATM and pRAD50 H-score, (**b**) comparison between the pRAD50 H-score and different types of ATM alterations across PDX models, (**c**) comparison of the ATM H-score and RAD51 foci score, (**d**) comparison between RAD51 foci scores and different types of ATM alterations across PDX models, and (**e**,**f**) represents the tATM H score (IHC method) correlation with best responses following AZD6738 monotherapy and combination with olaparib, respectively. Letters and numbers in brackets on the *x*-axis denote the ATM protein change and variant allele frequency (VAF), respectively. * Indicates the truncating ATM alteration and fs indicates a frameshift mutation.

All PDX models were selected based on the mutation profile of *ATM* as evaluated by historical WES data. At the time of the initiation of the study, we started with 980 PDX models having ATM alteration based on WES available data from the Crown Biosciences and Champions Oncology contract research organisations (CROs). We re-analysed the WES data internally and selected models based on (1) the presence of predicted deleterious truncating nonsense (*), frameshift (fs), splice, and/or clinically relevant [34] recurrent *ATM* mutations and had variant allele frequency (VAF) data available; (2) excluded models, where possible, with co-occurring mutations in other main DDR genes such as *BRCA1* or *BRCA2* which may have confounded the results (Appendix A, Figure A1c), and (3) robust in vivo tumour growth characteristics (Figure 1a). We hypothesised that a deleterious mutation with a VAF of >0.5 would be more likely to predict biallelic mutations and enrichment for functionally impaired ATM proteins while VAF ≤0.5 is likely to be indicative of a monoallelic mutation (50% alleles mutated). Using this stringent criteria we were left with only 15 PDX models (~1.5%) with predicted deleterious *ATM* mutations that included frameshift (*n* = 4, VAF > 0.5), nonsense (*n* = 6, VAF > 0.5; *n* = 2, VAF ≤ 0.5), splice variant (*n* = 1, VAF >0.5), and predicted clinically relevant missense (*n* = 2, VAF >0.5) alterations and selected two additional *ATM* wild-type models as controls. Subsequently, mutations were independently confirmed for each model by WES of tumours from vehicle-treated animals when within a study (Table 2). Frameshift and nonsense mutations will be collectively referred to as truncating mutations. These models were split across multiple tumour types including lung cancer (*n* = 4, 2), non-small cell lung cancer (NSCLC), one small cell lung cancer (SCLC), one large cell neuroendocrine carcinoma of the lungs (LCNEC), colorectal cancer (CRC) (*n* = 3), head and neck squamous cell carcinoma (HNSCC) (*n* = 3), gastric cancer (GC) (*n* = 2), pancreatic cancer (*n* = 2), glioblastoma (GBM) (*n* = 1), liver cancer (*n* = 1), and ovarian cancer (*n* = 1). The limited number of models available did not allow meaningful analysis by tumour type. CTG-0166 and CTG-0198 were derived from pre-treated patients (treatment unknown) whilst CTG-0149, CTG-0776, and CTG-0828 were derived from the treatment of naïve patients. For the rest of the models, patient treatment history was not available (Appendix A, Table A1).

To assess the baseline total (tATM) protein expression, we performed IHC staining (Figure 1b,c). We used IHC H scores of less than five and more than five to define ATM expression in these models as low and high tATM H scores, respectively. The IHC H score of five was a sufficiently low cut-off to identify complete protein loss while accounting for any background staining. LI6622 (splice site mutation) had the highest level of ATM protein expression across the PDX panel, with a mean tATM H score of 95. Two models with missense mutations, CR2506 (R3008H) and CR3280 (E2444k), also expressed ATM protein with mean H scores of 22 and 13, respectively. As expected, both *ATM* WT models (CTG-149 and CTG-0776) expressed tATM with H scores > 50. There was only one model (out of 7), CTG-1140, with a truncating mutation and VAF > 0.5 that expressed high amounts of ATM protein with an H score > 50. Interestingly, CTG-1140 had a VAF of 0.73 which could suggest an ATM copy number variation (tetraploid) in this model with only three of four copies mutated which may account for the ATM protein expression, although this was not confirmed. Two models (out of 5), CTG-0166 and CTG-0198, with truncating *ATM* mutations but with VAF ≤ 0.5 (likely monoallelic) also had high tATM IHC H scores of >50. The remaining models had only negligible ATM protein expression with low tATM IHC H scores of <5 (Figure 1c). We observed a clear enrichment of truncating *ATM* mutations with low tATM protein expression in 9 of 12 models (75%). By further stratifying the truncating mutation by VAF, we found that six of seven models (86%) with truncating VAF of >0.5 had low levels of tATM protein whereas three of five models (60%) with a truncating VAF of ≤0.5 expressed high tATM levels. Models with missense (E244K and R3008H) and splice-site mutations (I790I) did not associate with ATM protein loss (Figure 1c). The splice-site mutation has subsequently been reported as benign which could account for the presence of ATM protein in the LI6622 model [34,35].

**Figure 2:**
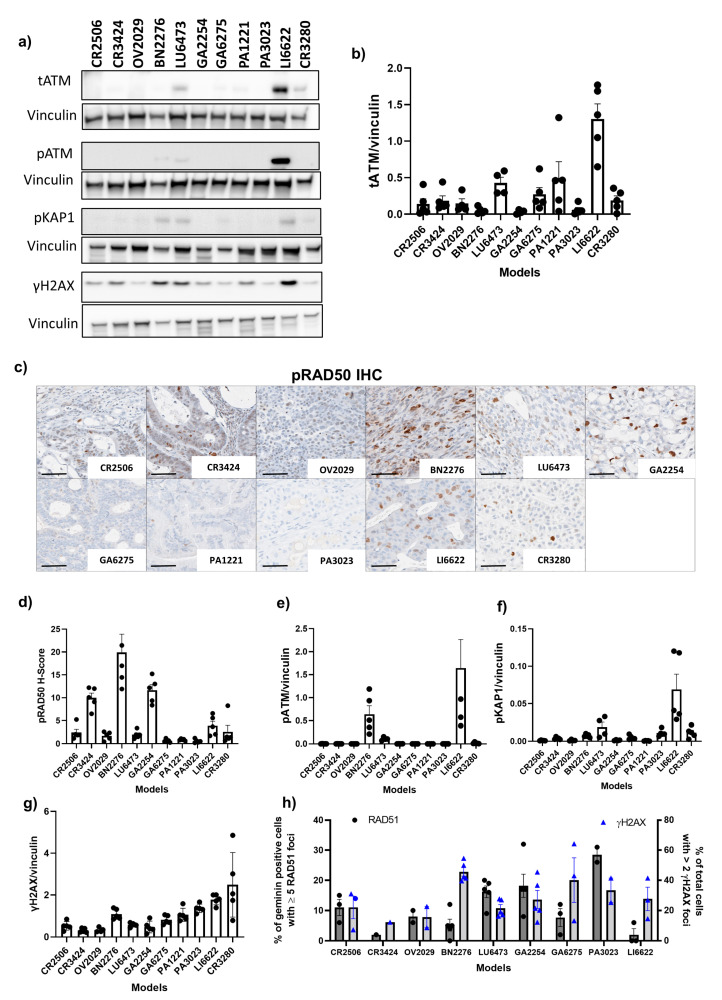
Baseline expression of downstream markers of ATM and RAD51 foci scores in PDX models. (**a**) Representative Western blot analysis for tATM, pATM, pKAP1, and γH2AX expression in 11 PDX models. The uncropped blots are shown in File S1. (**b**) represents the average expression of tATM normalised to vinculin by Western blot analysis, (**c**) representative IHC images of pRAD50 IHC in 11 PDX models (scale bar represents 50 µm), (**d**) represents the mean H score of pRAD50, (**e**–**g**) represents the average expression of pATM, pKAP1, and γH2AX normalised to vinculin, respectively, and (**h**) represents the baseline RAD51 foci score and γH2AX staining by immunofluorescence (IF) across 9 models. Data are represented as the mean ± SEM with *n* = 5 for CR2506, CR3424, BN2276, GA6275, PA1221m and PA3023, *n* = 4, for OV2029, LU6473, and GA2254 for Western blot analysis. For RAD51 and γH2AX IHC quantification, samples that presented a low RAD51 score (<5 foci per cell) and low γH2AX score (<10% of total cells with ≥2 foci) were considered not evaluable due to the low levels of DNA damage. For detailed Western blot analysis and IF, refer to the methods section.

In addition to IHC evaluation, we used Western blot analysis to assess tATM protein expression (Figure 2a,b) and enable the assessment of the full-length protein using a different antibody. tATM protein expression was undetectable in BN2276, GA2254, and PA3023 models which was consistent with IHC data. LI6622 showed strong expression of ATM which was consistent with high expression by IHC. However, in some models, the tATM protein expression profile obtained by Western blot analysis differed slightly from that obtained by IHC analysis. Western blot data showed low levels but clearly detectable expression of tATM in LU6473, PA1221, and GA6275 (Figure 2b) whereas IHC analysis indicated very low or undetectable ATM protein in these models. Of note, the IHC antibody for tATM binds to the serine 1981 region [36] whereas the Western blot antibody for tATM binds to the c-terminal region (aa 2550–3100) of the ATM protein (Appendix A, Figure A1b).

To better understand the functional relevance of *ATM* mutations and expression, we conducted more in-depth baseline profiling for downstream protein markers of ATM signalling in 11 evaluable *ATM* mutant models. We looked at the baseline expression of phosphorylated RAD50 on Ser-635 (pRAD50) which is a direct downstream target of ATM and surrogate biomarker of ATM/ATR kinase activity by IHC (Figure 2c,d) [37]. The baseline pRAD50 expression was more evident than tATM expression across the models. BN2276 showed the highest level of pRAD50 protein expression, with a mean H score of 20. GA2254, CR3424, OV2029, LI6622, CR2506, CR3280, and LU6473 all expressed pRAD50 at various levels. No clear relationship between baseline tATM and pRAD50 protein expression was observed by IHC (Appendix A Figure A3a). Furthermore, low baseline pRAD50 levels did not predict *ATM* mutations (Appendix A Figure A3b).

We also evaluated the baseline expression of other targets of ATM kinase activity, autophosphorylation on Ser-1981 (pATM) (Figure 2a,e) and phosphorylated KAP1 on Ser-824 (pKAP1) (Figure 2a,f), and γH2AX (Figure 2a,g) as a marker of DNA breaks/damage by Western blot analysis. Only BN2276, LU6473 (both truncating, VAF < 0.5), and LI6622 (splice site) expressed pATM and pKAP1, suggesting potentially active on-going ATM and/or DNA damage-dependent signalling in these models. All models expressed γH2AX, suggesting some level of endogenous DNA damage at the baseline. Interestingly, LI6622 displayed markedly higher levels of baseline pATM, pKAP1, and γH2AX (as well as tATM) than all other models, which may indicate high intrinsic genomic instability and active DDR signalling. CR3280 (missense) and PA3023 (truncating, VAF > 0.5) both expressed low but detectable levels of pKAP1 but lacked pATM expression. This could potentially mean that in the absence of ATM-dependent signalling, other kinases, for example ATR [37] or DNA-PK, could phosphorylate KAP1 or RAD50 in some models and highlights the heterogeneity and complexity in DDR-signalling.

RAD51 nuclear foci has been previously demonstrated as a functional marker of HRR [38] and predicted PARPi activity in pre-clinical models [39]. Therefore, to further characterise these models at the baseline, we used RAD51 foci in tumour cells at the S-G2 phase of the cell cycle (geminin positive cells) by immunofluorescence (IF) to assess the HRR functionality. In addition, we used γH2AX by IF to assess DNA damage and confirm that RAD51 foci positive cells had intrinsic DNA damage to activate HRR pathway. Overall, at baseline, RAD51 foci was detectable in all models, suggesting that HRR was functional in these models (Figure 2h). In addition, there was no relationship between ATM expression or mutation type with RAD51 scores in these models (Appendix A Figure A3c,d). We were unable to assess the HR mutational signatures as mutational signatures were developed using whole genome sequencing and we only had whole exome sequencing data for these models.

### 3.2. Antitumour Response to DDRi Agents in PDX Models with ATM Mutations

The 11 evaluable PDX models characterised ATM alterations were used as a platform to determine whether ATM status could be related to the antitumour activity of DDRi agents. We used the DDR agents AZD6738 (ATRi), AZD7648 (DNA-PKi), and olaparib (PARPi) which have previously been reported to show enhanced activity in ATM-deficient models [27,29]. This included both monotherapy and combination therapy at biologically active doses and schedules of the DDRi agents.

**Figure 3:**
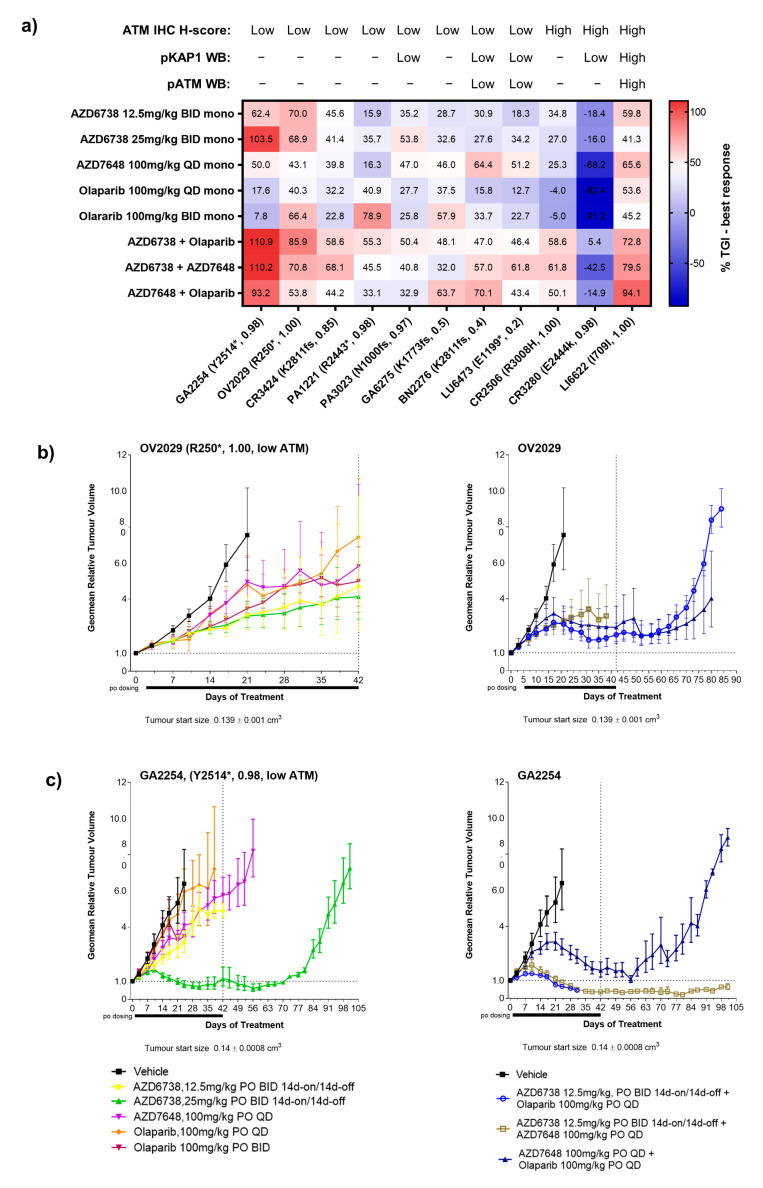
Antitumour responses in PDX models following treatment with DDR agents. (**a**) Heat map of the best response of DDRi agents both as a monotherapy and combination therapy in 11 PDX models. (**b**,**c**) Examples of tumour growth curves in GA2254 and OV2029 PDX models with Y2514* and R250* truncating ATM mutation, respectively, following monotherapy and combination therapy with DDR agents. AZD6738 was used at 12.5 mg/kg BID (7ON 7OFF) in combination therapy with olaparib or AZD7648 at 100 mg/kg QD continuous. Tumour growth curves represent the geometric relative tumour volume ± SEM (*n* = 5, per treatment arm). Dotted lines in tumour growth curves represent the end of the dosing period. The best response was calculated as the maximum % tumour growth inhibition (TGI) following a minimum 14 days of tumour growth for each respective model. Letters and numbers in brackets refer to the ATM protein change and variant allele frequency, respectively. * Indicates the predicted truncating ATM mutation and fs indicates a frameshift mutation. For the ATM status, models were labelled as low (<5) and high (>5) based on the IHC ATM H score. For the pATM and pKAP1 expression profiles, models were classed as low expressing relative to the expression level of these markers in LI6622 (pATM/pKAP1 high).

**Figure 4:**
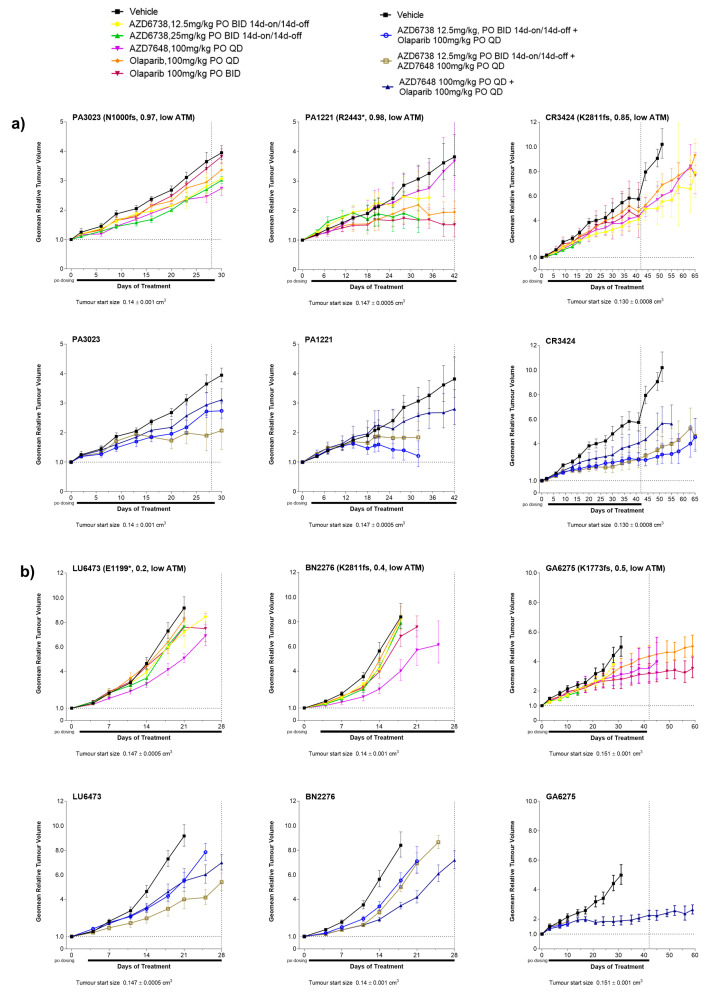
Antitumour responses in PDX models with truncating ATM mutations and low ATM IHC H scores (less than 5). (**a**) Representative tumour growth curves in PDX models with ATM truncating mutations with a variant allele frequency of 0.5 and above and (**b**) representative tumour growth curves in PDX models with ATM truncating mutation with an allele frequency of 0.5 and below (monoallelic) following treatment with DDR agents. AZD6738 was used at 12.5mg/kg BID (7ON 7OFF) in combination therapy with olaparib or AZD7648 at 100 mg/kg QD. Tumour growth curves represent the geometric relative tumour volume ± SEM (*n* = 5, per treatment arm). Dotted lines in tumour growth curves represent the end of the dosing period. Letters and numbers in brackets represent the ATM protein change and variant allele frequency, respectively. * Indicates the predicted truncating ATM mutation and fs indicates a frameshift mutation. For the ATM status, models were labelled as low (<5) and high (>5) based on the IHC ATM H score.

**Figure 5:**
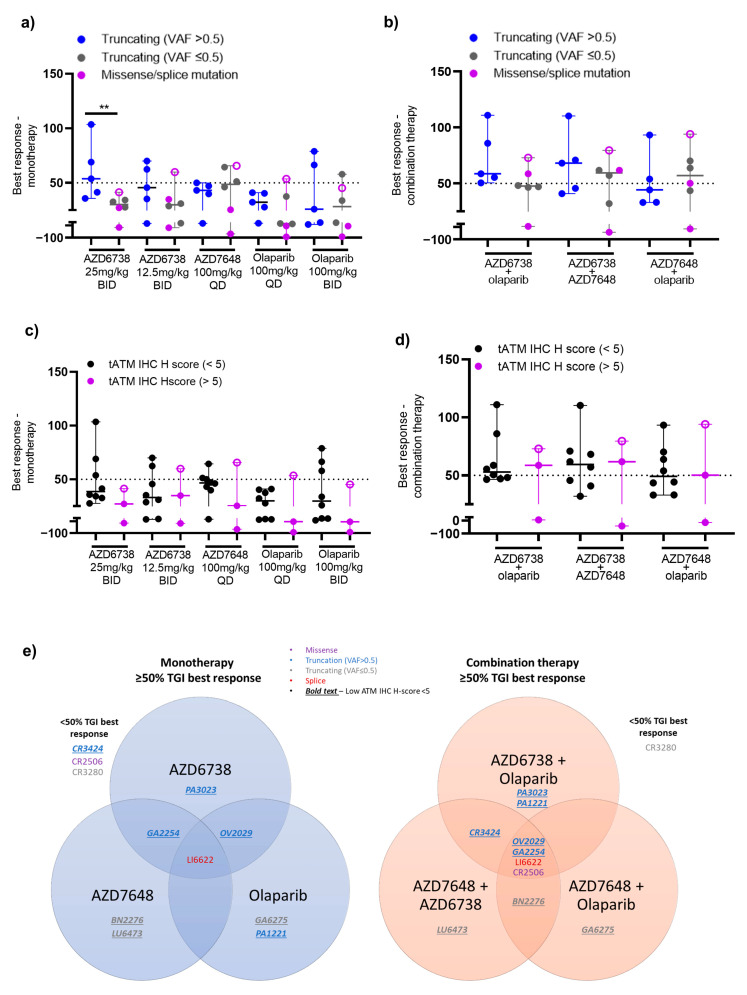
Correlation of the best responses of DDR agents and tATM assessment methods in 11 PDX models. (**a**,**b**) Median % TGI best response for monotherapy and combination therapy of DDR agents across models grouped according to ATM biallelic truncating mutation (VAF > 0.5) and ATM monoallelic (VAF ≤ 0.5)/missense/splice-site mutation, respectively; (**c**,**d**) Median % TGI best response for monotherapy and combination therapy of DDR agents across models grouped according to low (<5) or high (>5) tATM IHC H score, respectively, where each dot represents one model and LI6622 is represented as an open circle; (**e**) Venn diagram showing the best response across models with monotherapies and combination therapies with different DDRi agents. AZD6738 was used at 12.5 mg/kg BID (7ON 7OFF) in combination therapy with olaparib or AZD7648 at 100 mg/kg QD. The best response was calculated as the maximum % tumour growth inhibition (TGI) following a minimum of 14 days of tumour growth for each respective model. Horizontal dotted lines indicate a best response of 50% TGI. Non-parametric Mann–Whitney test pairwise statistical analyses for all groups are shown in Appendix A Table A3 and Table A4. ** Indicates a *p*-value < 0.01, otherwise results are non-significant.

**Figure A4:**
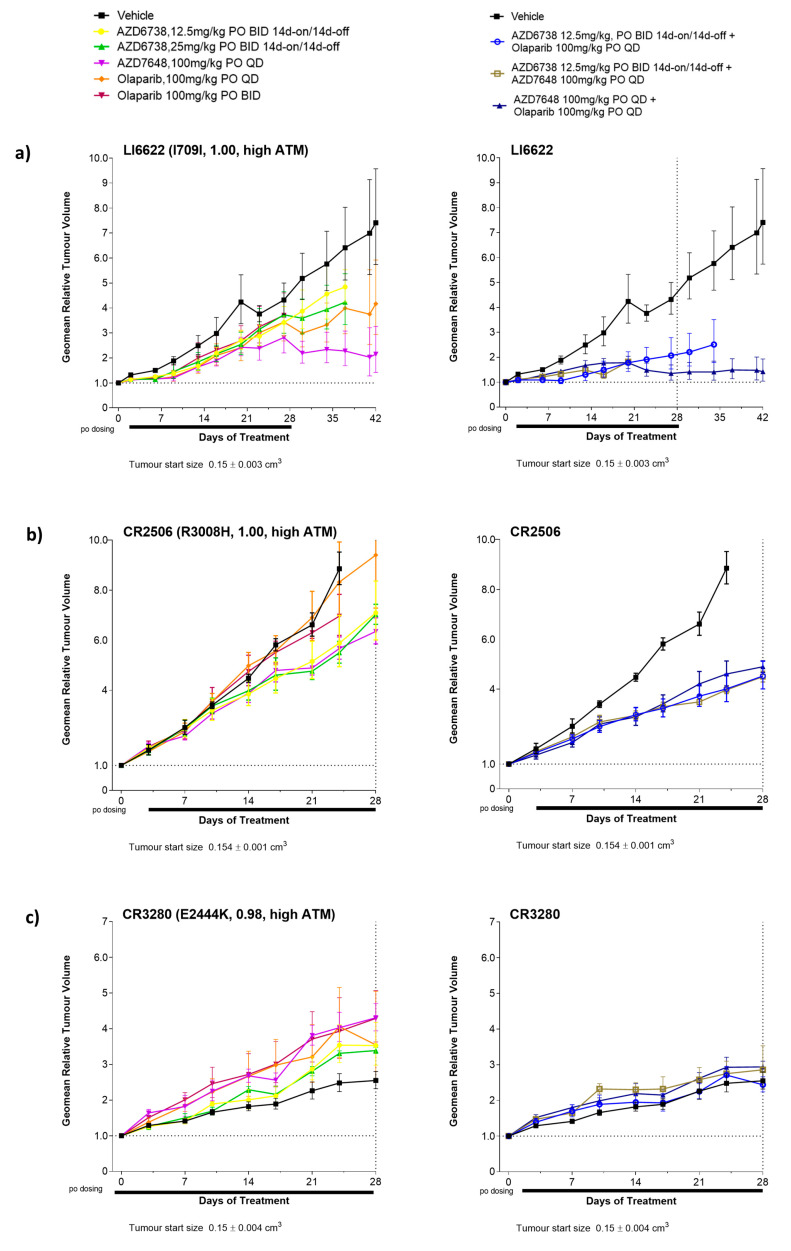
Antitumour responses in PDX models with non-truncating ATM mutations and high ATM IHC H scores (greater than 5). (**a**) Representative tumour growth curves in the presence of LI6622 with I709I ATM splice site mutations, (**b**) representative tumour growth curves in the CR2506 model with the R3008H missense ATM mutation, and (**c**) representative tumour growth curves in the CR3280 model with the E2444K missense ATM mutation following treatment with DDRi agents. AZD6738 was used at 12.5 mg/kg BID (7ON 7OFF) in combination therapy with olaparib or AZD7648 at 100 mg/kg QD. Tumour growth curves represent the geometric relative tumour volume ± SEM (*n* = 5, per treatment arm). Dotted lines in tumour growth curves represent the end of the dosing period. Letters and numbers in brackets indicate ATM protein change and variant allele frequency, respectively. For the ATM status, models were labelled as low (<5) and high (>5) based on the IHC ATM H score.

When used as monotherapy, all DDRi agents showed some degree of response across models with a trend for enrichment of activity for those models with truncating *ATM* mutation and VAF > 0.5 (Figure 3a). Overall, across all 11 PDXs regardless of the nature of the ATM alteration, AZD6738, AZD7648, and olaparib monotherapy treatments (at any dose level) all achieved a minimum best response of 50% tumour growth inhibition (TGI) in 4 out 11 models (36%) albeit the responses were not in the same models. Further stratification of models by truncating *ATM* mutations (*n* = 8; all tATM IHC H score low) showed that AZD6738 monotherapy achieved a minimum of 50% TGI best response in three (GA2254, OV2029, and PA3023) of five models with a VAF >0.5 (60%) (Figure 3a–c and Figure 4a for tumour growth curves) and in none of the three models with a VAF ≤ 0.5 (0%) (Figure 3a and Figure 4b for tumour growth curves). AZD7648 monotherapy achieved at least 50% TGI in 1 (GA2254) of the 5 models with a VAF > 0.5 (20%) and two (BN2276 and LU6473) of the three models (40%) with a VAF ≤ 0.5 for *ATM* truncating mutation. Olaparib monotherapy showed at least 50% TGI activity as the best response in OV2029, GA6275, and PA1221 models. These models had *ATM* truncating mutations with PA1221 and OV2029 having VAF of >0.5 (two out of five, 40%) whereas GA6275 had a VAF of ≤0.5 (one out of three, 33%). Interestingly, the LI6622 model showed a >50% TGI best response to all three DDRis as a monotherapy. LI6622 contained an ATM slice site mutation and high baseline tATM protein IHC H score and was unique in that it also displayed high baseline pATM, pKAP1, and γH2AX (Figure 2 and Figure 3a and Appendix A, Figure A4 for growth curves). None of the models with missense *ATM* alterations (all high tATM IHC H score) (Figure 3a and Figure 5b,c) achieved a minimum of 50% TGI as the best response to monotherapy with any of the DDRi agents. AZD6738 was the only DDRi agent acting as a monotherapy that achieved tumour regression (>100% TGI) in the GA2254 model (Figure 3a,c).

When used in combination, the number of antitumour responses (>50% TGI) was increased compared to monotherapy treatments. AZD6738 combined with olaparib achieved a minimum best response of 50% TGI in all five models with *ATM* truncating mutations and a VAF > 0.5 (100%) (Figure 3a and Figure 4a) but in none of the three models with a VAF ≤0.5 for truncating *ATM* mutations (0%) (Figure 3a and Figure 4b). AZD7648 combined with AZD6738 or olaparib achieved a minimum of 50% TGI in three (GA2254, OV2029, and CR3424; 60%) and two (GA2254 and OV2029; 40%) models, respectively, of the five models with a VAF > 0.5 (Figure 3a and Figure 4a) for truncating the *ATM* mutation. In addition, AZD7648 with AZD6738 or olaparib combinations achieved a minimum of 50% of the TGI best response in one model (BN2276) and AZD7648 with olaparib in another model (LU6473) of the three models with a VAF of ≤0.5 (Figure 3a and Figure 4b) for the truncating *ATM* mutation (33%). For all combination therapies, CR2506, one of the two models with missense *ATM* alterations, achieved a minimum best response of 50% TGI (Figure 3a and Figure 5b). Similar to monotherapy, LI6622 showed activity with all combination therapies (Figure 3a and Figure 5a). GA2254 was the only model in which combination therapy drove tumour regression with AZD6738 plus olaparib or AZD6738 plus AZD7648 (Figure 3a,c). AZD7648 plus olaparib achieved a best response TGI of 93% in this model (Figure 3a). CR3280 (E2444k missense mutation VAF > 0.5, high tATM IHC H score) was refractory to all agents either as a monotherapy or in combination.

For monotherapies across models, the high dose of AZD6738 at 25mg/kg BID achieved a significantly better median best response (54% TGI, *p* = 0.009) in models with biallelic truncating *ATM* mutations (VAF > 0.5) compared to models with monoallelic truncating (VAF ≤ 0.5) and missense/splice-site *ATM* mutation (Figure 5a and Appendix A, Table A2). The rest of the DDRi agents did not show any significant differentiation in median best response between models with biallelic truncating and monoallelic/missense/splice-site *ATM* mutations. The combination of AZD6738 and olaparib (Appendix A, Table A2) was able to achieve a significantly better median best response (59% TGI) across biallelic *ATM* mutant (VAF > 0.5) models compared to monoallelic *ATM* mutant models (VAF ≤ 0.5). In response, no other combinations showed differentiation across the PDX models with different *ATM* alterations (Figure 5b and Appendix A, Table A2).

### 3.3. Correlation of the Best Response in PDX Models with ATM Protein Expression

We used IHC to determine tATM expression and also evaluated pATM and pKAP1 expression by Western blot analysis as markers of the ATM signalling pathway at the baseline for all 11 ATM mutant PDX models. Therefore, we wanted to understand which protein level assessment may be a better predictor of sensitivity to DDRi drugs. We used XY plots to compare the level of biomarker expression and the best TGI response.

For AZD6738 as a monotherapy, three out of eight models (GA2254, OV2029, and PA3023, 38%) with low tATM IHC H scores achieved a minimum best response of 50% TGI while none of the three models (0%) with high tATM IHC H scores achieved a 50% TGI response (Appendix A, Figure A3e). AZD6738 combination therapy with olaparib showed similar patterns in terms of the best response when compared with tATM expression (Appendix A, Figure A3f) but with four additional models now exceeding the 50% TGI response, two with low tATM IHC H scores (CR3424, PA1221; five out of eight, 63% with low tATM), and two with high tATM (CR2506, LI6622; two out of three, 67% with high tATM). Overall, 7 out 11 models (64%) showed a minimum 50% TGI best response with a combination of AZD6738 and olaparib. There was a numerical trend for the enrichment of responses in models with low tATM H scores by IHC for AZD6738 monotherapy (38% low vs. 0% high) but no differential for a combination where high tATM models showed >50% TGI responses (63% low vs. 67% high) but with the caveat of a low number of models. GA2254 and OV2029 consistently showed the greatest sensitivity. LI6622 was a clear outlier as this model was sensitive to AZD6738 as a monotherapy and responded to combination therapy with olaparib despite having a high level of tATM expression. In addition, CR2506, which had high tATM expression, showed activity in the combination treatment arm with a best response TGI of >50%, thus suggesting that combination activity may extend beyond low ATM protein expression. CR3280, expressing high tATM levels, showed no response to either monotherapy or combination therapy.

**Figure A5:**
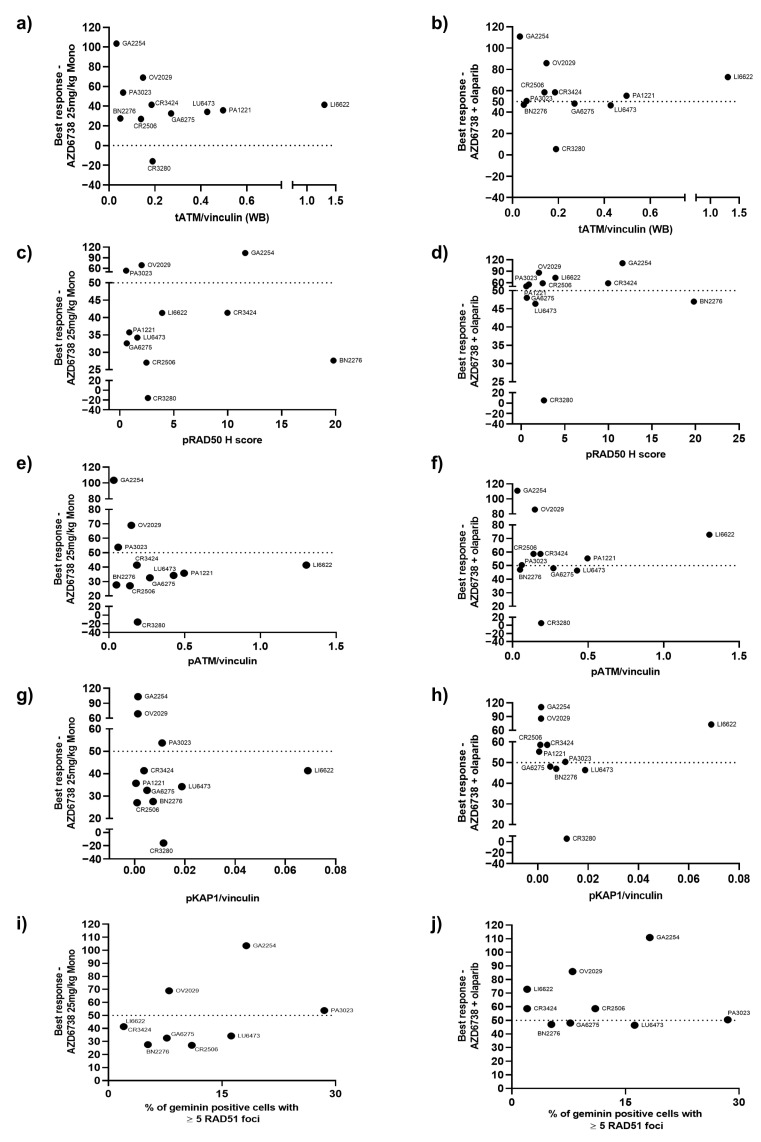
Correlation of the best response of DDR agents with tATM expression by Western blot analysis and the expression of downstream signalling marker scores of ATM and RAD51 in PDX models. (**a**,**b**) Correlation of tATM expression with the best response following AZD6738 monotherapy and combination with olaparib, respectively, (**c**,**d**) correlation of pRAD50 expression by IHC with the best response following AZD6738 monotherapy and combination with olaparib, respectively, (**e**,**f**) represents the correlation of pATM expression with best response following AZD6738 monotherapy and combination with olaparib, respectively, (**g**,**h**) represents the correlation of pKAP1 expression with the best response following AZD6738 monotherapy and combination with olaparib, respectively, (**i**,**j**) represents the correlation of the RAD51 foci score with the best response following AZD6738 monotherapy and combination with olaparib, respectively. Values of the best response represent the % TGI for AZD6738 monotherapy at 25 mg/kg BID 7ON 7OFF and the combination at 12.5 mg/kg BID with 100 mg/kg olaparib QD. Horizontal dotted lines indicate the best response of 50% TGI.

We also examined the association of pRAD50 (IHC), tATM, pATM, and pKAP1 (Western blot analysis) baseline protein expression levels with the best response from AZD6738 as a monotherapy and in combination (Appendix A, Figure A5a–h). In addition, we wanted to check if there was any association with HRR status assessed by the RAD51 foci score and treatment response (Appendix A Figure A5i,j). None of these markers added to the segregation of response that we saw with the tATM IHC score at the baseline for these models. 

**Figure A6:**
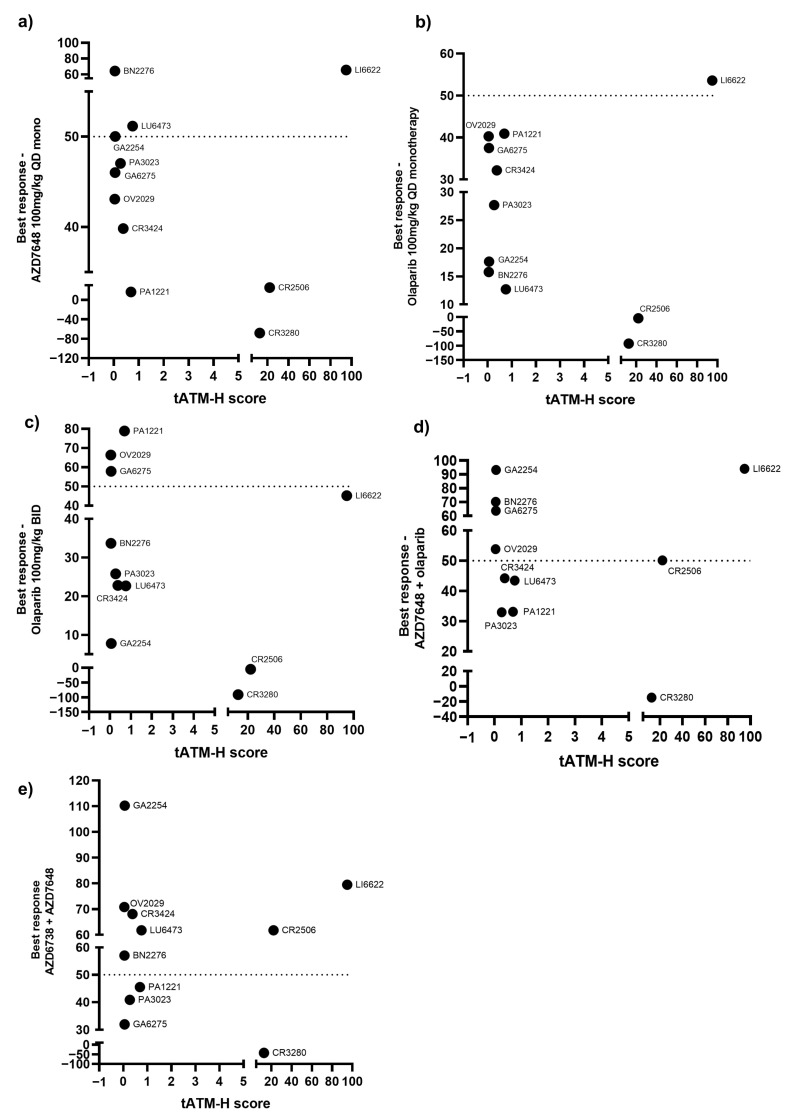
Correlation of the best response of DDR agents with the tATM H score in 11 PDX models. (**a**) AZD7648 monotherapy, (**b**,**c**) represents the correlation of the tATM H score with the best response following olaparib treatment at 100 mg/kg QD and 100mg/kg BID, respectively, (**d**) AZD7648 + olaparib, and (**e**) AZD7648 + AZD6738 represents the correlation of the tATM H score with the best response. For combinations, values of best responses represent the % TGI for AZD6738 at 12.5 mg/kg BID (7ON 7OFF) and olaparib at 100 mg/kg QD in combination with AZD7648 at 100 mg/kg QD. The best response was calculated as the maximum % tumour growth inhibition (TGI) following a minimum of 14 days of tumour growth for each respective model. Horizontal dotted lines indicate a best response of 50% TGI.

We further compared the best response of other DDRi agents with the baseline expression of tATM, as assessed by IHC (Figure 5c,d, Appendix A Figure A6). For monotherapy with AZD7648 at 100 mg/kg QD or olaparib at 100 mg/kg BID, three of the eight models with low tATM IHC H scores (38%) showed responses as did one out of the three models with high tATM (33%) for both agents. However, responsive models differed between agents (Figure 5e). For AZD7648 monotherapy, low tATM H score models BN2276, LU6473, and GA2254 (also responsive to AZD6738) showed > 50% TGI response while for olaparib monotherapy PA1221, OV2029 (also responsive to AZD6738), and GA6275 low tATM H score models responded, suggesting additional drivers of sensitivity in these models for each agent. Increased activity was observed for AZD7648 combination with olaparib, with 6 out of 11 (55%) of the total models achieving a minimum 50% TGI best response, with 4 models out of 8 with low tATM (GA2254, BN2276, GA6275, and OV2029, 50%), and 2 models out of 3 with high tATM (CR2506 and LI6622, 67%). AZD7648 in combination with AZD6738 showed 7 out of 11 (64%) total models with a minimum 50% TGI response, with 5 of 8 models with low tATM (GA2254, OV2029, CR3424, LU6473, and BN2276, 63%) and 2 of 3 models with high tATM (CR2506, LI6622, 67%) again responding.

The median best response TGI across all DDRi monotherapies was numerically higher in models with low ATM IHC H scores compared to the median TGI across models with high ATM H scores. However, due to low numbers of PDX models, this was not statistically significant and we could only see a trend towards a better response in models with low ATM H scores compared to models with high ATM H scores (Figure 5c and Appendix A, Table A3). Furthermore, there was no significant difference in the median best response TGI across all combination therapies between models with low and high ATM H scores, respectively (Figure 5d and Appendix A Table A3). When comparing monotherapies versus combination best responses, the only combination that showed significantly different mean TGIs as the best response (63% ± 8.3) from respective monotherapies was AZD6738 (38.7% ± 7, *p* = 0.001) and olaparib (28.4% ± 4, *p* = 0.007) across models with an ATM IHC H score of less than five (Appendix A, Table A4).

## 4. Discussion

Early clinical studies with DDRi agents showed some success in patients with ATM loss. For PARP inhibitors, the TOPARP-B phase 2 clinical study [40] and PROfound phase 3 study in patients with metastatic castration-resistant prostate cancer [41] included olaparib treatment in patients with *ATM* alterations and with other HRR gene mutations, leading to its approval by the U.S. Food and Drug Administration [8]. However, a double-blind and randomised study (GOLD) failed to show a statistically significant benefit of olaparib combined with paclitaxel over paclitaxel alone in patients with advanced gastric cancer with low ATM protein expression, as assessed by IHC [42]. For ATR inhibitors, a first-in-human study with the ATR inhibitor BAY1895244 [43] monotherapy in advanced solid cancers reported initial evidence of a benefit in patients with *ATM* alterations (mutation or protein loss); however, in the phase 1b expansion study, this activity declined and ATM protein loss was not predictive of a progression-free survival benefit [44]. In an early clinical first-in-human study of another ATR inhibitor, RP-3500, similar modest monotherapy responses were observed in patients with *ATM*-mutant tumours [45]. Interestingly, a trend was observed in that study for improved clinical benefits in biallelic versus monoallelic mutations. Therefore, it remains unclear whether ATM protein loss and/or mutations would effectively predict a response to DDRi agents.

In our *ATM* mutant PDX study, the majority of models (10 of 11) showed varying degrees of sensitivity to DDRi agents either as aa monotherapy or combination therapy. However, the only model (GA2254) in which DDRi agents drove tumour regression both as monotherapy and combination therapy had a low tATM H score. In addition, both models with *ATM* missense mutations, CR3280 and CR2506, were clearly characterised by high tATM levels and showed no activity of DDRi agents as a monotherapy; CR3280 was refractory to all treatments (Figure 3a and Figure 5c). Interestingly, LI6622, which harboured *ATM* splice site mutations, was characterised as a high tATM expressing protein but also by its high baseline DDR-signalling, showing sensitivity to DDRi agents both as a monotherapy and combination therapy (Figure 3a and Figure 5a). This finding suggests that sensitivity to DDRi agents could be independent of ATM, that other molecular factors could be driving a DDR, and the efficacy in this model. We evaluated molecular alterations by WES across pan-cancer genes of interest at the baseline in our PDX models (Appendix A, Figure A1c) and did not find any stand-out molecular signature that could be related to sensitivity to DDRi agents in these models. In addition, the HRR status, as assessed by RAD51 foci formation, did not correlate with the DDRi responses. Although RAD51 foci have been validated as functional markers of HRR and are predictive of PARPi responses [39], the assay has not been validated across different tumour types as represented by this cohort of PDX models. Furthermore, we did not observe any correlation between the RAD51 foci score and the *ATM* mutation or protein expression. This was not surprising as ATM is not considered as a core marker of the HRR pathway and because ATM knockout models could elicit only a modest response to PARPi in vitro compared to *BRCA* mutated cells [46].

Overall, there was some enrichment in the sensitivity to DDRi agents in models with biallelic (VAF > 5) truncating *ATM* mutations and ATM protein loss, especially in the combination setting. For example, AZD6738 monotherapy showed a minimum of 50% TGI as the best response in three of the eight models (38%) with low tATM IHC H scores whereas this increased to five of the eight models (63%) with the combination of AZD6738 with olaparib (Figure 5c,d). Across all DDRi monotherapies, six of eight models (75%) with *ATM* truncating mutations achieved a minimum best response of 50% TGI while all of these eight models (100%) achieved a minimum 50% TGI for combination therapies across all the DDRi agents. All of these models also had low tATM IHC H scores. Only one of the two models with missense *ATM* mutations showed activity across all DDR combinations. The only model with a splice-site *ATM* mutation (LI6622) had a high tATM IHC H score and high baseline pATM/pKAP1 signalling and showed activity across all the DDRi agents both as monotherapy and combination therapy. It is unknown as to whether the splice-site *ATM* mutation is related to or independent of the baseline DDR activation in this model. With the caveat of the low number of models, only *ATM* truncating mutations with a VAF of >0.5 and protein loss could enrich the DDRi sensitivity in these PDXs. Clinical investigation of *ATM* mutations and protein loss is currently being explored for AZD6738 as a monotherapy in the PLANETTE study [47].

## 5. Conclusions

The role of ATM in the DDR pathway is well established; however, how best to use ATM as a patient selection marker for better clinical outcomes remain unclear. The challenge lies in assessing how ATM mutations and loss of protein expression could help in predicting treatment responses to DDRi. Here, we have shown that biallelic (high VAF) truncating *ATM* mutations correlated with a loss of protein expression. In addition, the maximum responses to DDRi were observed in models with biallelic truncating mutations/low tATM IHC H scores; although, responsive models sometimes differed between DDRi agents suggesting additional features may contribute to their activity. Therefore, *ATM* mutant/protein-loss tumours likely represent a heterogenous population. The limitations of this study were the low number of evaluable ATM PDX models and, subsequently, the inability to assess potential tumour type differences. Looking at the type of *ATM* mutation with VAF and confirming tATM expression at the baseline by IHC along with an understanding of the molecular phenotype of tumours could aid in mapping treatment responses to ATM status for certain patient populations.

## Figures and Tables

**Table 1:** Study design.

Treatment Group	Comments
Vehicle control	
AZD6738, 12.5 mg/kg PO BID (14 days on, 14 days off)	BID dosed 8 h apart
AZD6738, 25 mg/kg PO BID (14 days on, 14 days off)	BID dosed 8 h apart
AZD7648, 100 mg/kg PO QD continuous	
Olaparib, 100 mg/kg PO QD continuous	
Olaparib, mono 100 mg/kg PO BID	
AZD7648, 100 mg/kg PO QD continuous	
AZD6738, 12.5 mg/kg PO BID (14 days on, 14 days off) + olaparib 100 mg/kg PO QD continuous	AZD6738 dosed 1 h after olaparib dose
AZD6738, 12.5 mg/kg PO BID (14 days on, 14 days off) + AZD7648, 100 mg/kg PO QD	AZD6738 dosed 1 h after AZD7648 dose
AZD7648, 100 mg/kg PO QD + olaparib, 100 mg/kg PO QD	Olaparib dosed 1 h after AZD7648 dose

**Table 2:** PDX model characterisation.

Model ID	Tumour Type	Protein Change	Mutation Type	ATM VAF Original (a)	ATM mRNA Expression [log2(Value + 1)]	tATM Mean IHC H Score	pRAD50 Mean IHC H Score	pATM/Vinc WB Signal (b)	pKAP1/Vinc WB Signal (b)
CR2506	Colorectal (ADC)	R3008H	Missense	1 (1)	10.28	22.2	2.45	0.002 (0.13)	0.001 (1.5)
CR3424	Colorectal (ADC)	K2811fs	Frameshift	1 (0.85)	NA	0.38	9.97	0.002 (0.11)	0.004 (5.4)
OV2029	Ovarian	R250 *	Nonsense	1 (1)	NA	0.05	1.70	0 (0)	0.001 (1.9)
BN2276	Glioblastoma	K2811fs	Frameshift	0.63 (0.4)	NA	0.05	19.90	0.64 (39)	0.007 (10.7)
LU6473	Lung	E1199 *	Nonsense	0.53 (0.2)	10.93	0.76	1.98	0.12 (6.6)	0.019 (27.3)
GA2254	Gastric	Y2514 *	Nonsense	0.89 (0.98)	NA	0.06	11.63	0.002 (0.11)	0.001 (2)
GA6275	Gastric (ADC)	K1773fs	Frameshift	0.57 (0.5)	11.33	0.05	0.64	0 (0)	0.005 (7.2)
PA1221	Pancreatic (ADC)	R2443 *	Nonsense	0.97 (0.98)	9.84	0.69	0.87	0 (0)	0.001 (0.9)
PA3023	Pancreatic (ADC)	N1000fs	Frameshift	0.95 (0.97)	NA	0.27	0.58	0 (0)	0.011 (16)
LI6622	Liver	I709I	Splice region	1 (1)	10.73	94.87	3.92	1.64 (100)	0.069 (100)
CR3280	Colorectal (ADC)	E2444K	Missense	1 (0.98)	10.55	12.68	2.60	0.016 (1)	0.012 (16.8)
CTG 0828	Large-cell lung (ADC)	E473 *	Nonsense	1	1	2.16	10.6	NA	NA
CTG 1140	Head and neck (SCC)	R35 *	Nonsense	0.73	NA	80.91	NA	NA	NA
CTG 0166	Lung (SCC)	W3055 *	Nonsense	0.44	NA	55.49	10.4	NA	NA
CTG 0198	Small-cell lung	W1858 *	Nonsense	0.42	NA	78.82	NA	NA	NA
CTG 0149	Head and neck	NA	NA	NA	100	71.93	NA	NA	NA
CTG 0776	Head and neck	NA	NA	NA	100	51.47	NA	NA	NA

ADC, adenocarcinoma; NA, not applicable; SCC, squamous cell carcinoma; IHC, immunohistochemistry; VAF, variant allele frequency. (a) Confirmation of ATM VAF from internal whole exome sequencing (WES) where WB refers to Western blot and (b) the % expression relative to LI6622; * truncating alterations.

## Data Availability

Data underlying the findings described in this manuscript are available on request and may be obtained in accordance with AstraZeneca’s data sharing policy described at https://astrazenecagrouptrials.pharmacm.com/ST/Submission/Disclosure and global publications standards policty described at https://www.astrazeneca.com/sustainability/resources.html#policies-and-position-statements-0.

## Supplementary Materials

The following supporting information can be downloaded at: https://www.mdpi.com/article/10.3390/cancers15164195/s1, File S1: Full pictures of the Western blots and the densitometry scans.

## Appendix A

**Table A1:** Clinical summary of PDX models.

Model ID	Cancer Type	Subtype	Biopsy Site	Stage	Treatment History	Age	Gender	Ethnicity
CR2506	Colorectal Cancer	ADC	Colorectum	NA	NA	74	Female	Asian
CR3424	Colorectal Cancer	ADC, mucinous	Primary	NA	NA	NA	Female	Asian
OV2029	Ovarian Cancer	Serous ADC	NA	NA	NA	NA	Female	Caucasian
BN2276	Brain Cancer	Glioblastoma	Brain	NA	NA	65	Female	Asian
LU6473	Lung Cancer	LCNEC	NA	NA	NA	84	Female	Caucasian
GA2254	Gastric Cancer	ADC	Primary	cT4aN3M1	NA	67	Female	Asian
GA6275	Gastric Cancer	ADC	Primary	NA	NA	75	Male	Asian
PA1221	Pancreatic Cancer	Ductal ADC	Pancreas	NA	NA	67	Female	Asian
PA3023	Pancreatic Cancer	Ductal ADC	Pancreas	NA	NA	82	Female	Asian
LI6622	Liver Cancer	HCC	Liver, right lobe	NA	NA	34	Male	Asian
CR3280	Colorectal Cancer	ADC	Lymph node	NA	NA	63	Female	Asian
CTG 0828	Lung	NSCLC	Lymph node	II	Naive	81	Female	Caucasian
CTG 1140	Head and neck	SCC	Salivary gland	II	NA	70	Male	Caucasian
CTG 0166	Lung	NSCLC	Lung	I	Pre-treated	63	Female	Caucasian
CTG 0198	Lung	SCLC	Lung	III	Pre-treated	67	Male	NA
CTG 0149	Head and neck	SCC	Skin	III	Naive	77	Male	Caucasian
CTG 0776	Head and neck	SCC	Tongue	II	Naive	72	Female	Caucasian

ADC—adenocarcinoma; NA—not applicable; SCC—squamous cell carcinoma; NSCLC—non-small cell lung cancer; SCLC—small cell lung cancer; LCNEC—large cell neuroendocrine carcinoma.

**Table A2:** Best responses across models based on ATM mutations.

		Median Best Response as % TGI (95% CI)				* *p*-Value		
Treatment Group	Dose (mg/kg) and Schedule	ATM Truncating VAF > 0.5 (*n* = 5) A	ATM Truncating VAF ≤ 0.5 (*n* = 3) B	ATM Missense (*n* = 3) C	ATM Truncating VAF ≤ 0.5 + Missense (*n* = 6) B + C	A vs. B	A vs. C	A vs. B + C
AZD6738	25 BID 14ON 14OFF	54 (27, 94)	33 (23, 40)	27 (−20, 60)	30 (3, 46)	0.03	0.03	0.009
AZD6738	12.5 BID 14ON 14OFF	46 (19, 73)	29 (9, 43)	35 (−74, 125)	30 (−1, 53)	0.3	0.4	0.2
AZD7648	100 QD continuous	43 (23, 56)	51 (30, 77)	25 (−163, 178)	49 (−22, 84)	0.1	0.8	0.5
Olaparib	100 QD continuous	32 (20, 44)	16 (−12, 56)	−4 (−197, 168)	14 (−50, 58)	0.3	0.6	0.3
Olaparib	100 BID continuous	26 (2, 78)	34 (−7, 83)	−5 (−188, 154)	28 (−46, 68)	>0.99	0.3	0.4
AZD6738 + olaparib	12.5 BID 14ON 14OFF + 100 QD continuous	59 (40, 104)	47 (45, 49)	59 (−43, 134)	48 (23, 70)	0.03	0.8	0.1
AZD6738 + AZD7648	12.5 BID 14ON 14OFF + 100 QD continuous	68 (33, 101)	57 (11, 90)	62 (−131, 197)	59 (−5, 88)	0.4	0.8	0.4
AZD7648 + olaparib	100 QD + 100 QD continuous	44 (21, 82)	64 (25, 94)	50 (−93, 179)	57 (12, 90)	0.6	>0.99	0.7

* *p*-value = non-parametric Mann–Whitney test, median best response % TGI compared between models with different ATM mutations for each treatment group, respectively. BID—twice-daily dosing, QD—once-daily dosing.

**Table A3:** Best response across models based on ATM expression by IHC.

		Median Best Response as % TGI (95% CI)		
Treatment Group	Dose (mg/kg) and Schedule	ATM IHC H Score < 5 (*n* = 8)	ATM IHC H Score > 5 (*n* = 3)	* *p*-Value
Monotherapy				
AZD6738	25 BID 14ON 14OFF	39 (28, 104)	27 (−16, 41)	0.1
AZD6738	12.5 BID 14ON 14OFF	33 (16, 70)	35 (−18, 60)	0.8
AZD7648	100 QD continuous	47 (16, 64)	25 (−68, 66)	0.6
Olaparib	100 QD continuous	30 (13, 41)	−4 (−92, 54)	0.5
Olaparib	100 BID continuous	30 (8, 79)	−5 (−91, 45)	0.2
Combinations				
AZD6738 + olaparib	12.5 BID (14ON 14OFF) + 100 QD continuous	53 (46, 111)	58 (5, 73)	>0.99
AZD6738 + AZD7648	12.5 BID (14ON 14OFF) + 100 QD continuous	59 (32, 110)	62 (−43, 80)	>0.99
AZD7648 + olaparib	100 QD + 100 QD continuous	49 (33, 93)	50 (−15, 94)	>0.99

* *p*-value = non-parametric Mann–Whitney test, best response % TGI compared between models with ATM IHC score <5 and ATM IHC H score >5 for each treatment group, respectively. BID—twice-daily dosing, QD—once-daily dosing.

**Table A4:** Comparison of best response between monotherapy and combination.

Treatment Group	Dose (mg/kg) and Schedule	Mean % TGI ± SEM		* *p*-Value Combo vs. Mono	
		ATM IHC H Score < 5 (*n* = 8)	ATM IHC H Score > 5 (*n* = 3)	ATM IHC H Score < 5	ATM IHC H Score > 5
AZD6738	25 BID 14ON 14OFF	50 ± 9	18 ± 41	NA	NA
AZD6738	12.5 BID 14ON 14OFF	38 ± 7	25 ± 23	NA	NA
AZD7648	100 QD continuous	45 ± 5	8 ± 40	NA	NA
Olaparib	100 QD continuous	28 ± 4	−14 ± 42	NA	NA
Olaparib	100 BID continuous	40 ± 9	−17 ± 40	NA	NA
AZD6738 + olaparib	12.5 BID 14ON 14OFF + 100 QD continuous	63 ± 8.3	46 ± 21	vs. AZD6738: 0.001 vs. olaparib: 0.007	vs. AZD6738: 0.03 vs. olaparib: 0.1
AZD6738 + AZD7648	12.5 BID 14ON 14OFF + 100 QD continuous	61 ± 8.6	33 ± 38	vs. AZD6738: 0.001 vs. AZD7648: 0.1	vs. AZD6738: 0.7 vs. AZD7648: 0.06
AZD7648 + olaparib	100 QD + 100 QD continuous	54 ± 7.3	43 ± 32	vs. AZD7648: 0.2 vs. olaparib: 0.03	vs. AZD7648: 0.06 vs. olaparib: 0.2

* *p*-value—paired *t* test, mean best response as % TGI compared between models with ATM IHC score < 5 and ATM IHC H score > 5 for each treatment group, respectively. Comparison conducted at equivalent doses of each compound between combination and respective monotherapy groups. BID—twice-daily dosing, QD—once-daily dosing, NA—not applicable.
