# Supplementary material for: Relevance of ATM Status in Driving Sensitivity to DNA Damage Response Inhibitors in Patient-Derived Xenograft Models
Source: Cancers (Basel). 2023 Aug 21;15(16):4195. doi: 10.3390/cancers15164195 (PMC10453052; doi:10.3390/cancers15164195)
Supplement: Supplementary file 1 [file cancers-15-04195-s001.zip › File S1 Supplementary data western blots.pdf]

AstraZeneca

PDX models
Western blot images

Adina Hughes / Ankur Karmokar

4th Feb 2022

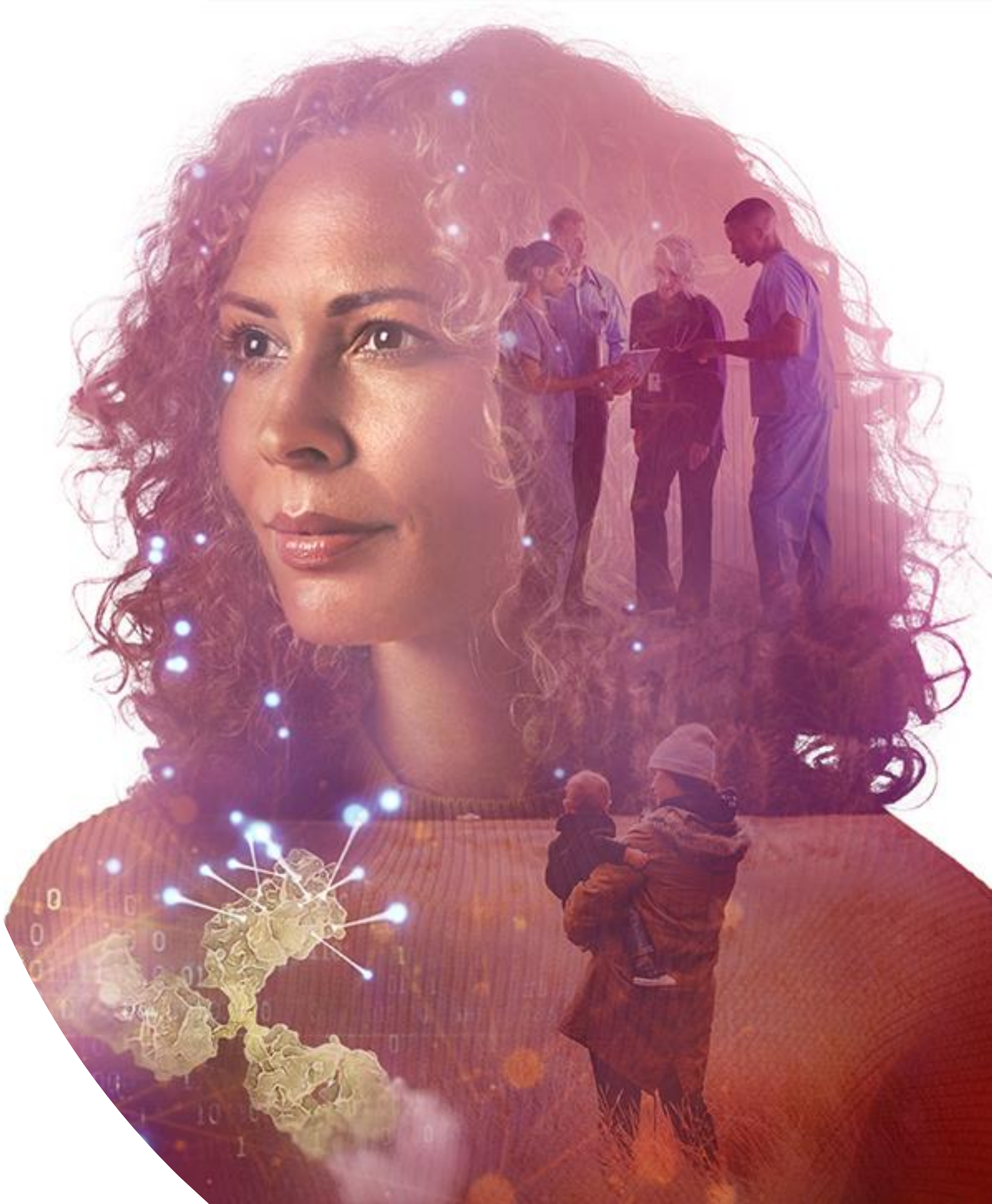

Western lysate prep & antibodies probed publication images

- Frozen tumour homogenised via fast prep in complete KDR lysis buffer
- 40µg ran in gels (Bis tris (BT) / MES 180V or Tris acetate (TA) 150V), iblot transfer BT 7min 20V or 10min TA
- Antibodies (primary O/N in 5% marvel in TBS/T & secondary 1hr RT)
- Cell line +IR 10Gy 2h lysate run as positive control for some DNA response proteins
- Imaged together per endpoint using WestDura on Sygnene Gbox (unsaturated images quantified)
- Data plotted as raw RLU (transformed), normalised vinculin

PDX model ID	Type
CR2506	CRC (ADC)
CR3424	CRC (ADC)
OV2029	Ovarian
PA1221	PAN (DC)
PA3023	PAN (DC)
GA2254	Ring-cell carcinoma
LI6622	Liver (HCC)
CR3280	CRC (ADC)
BN2276	GBM
LU6473	Lung (NEC)
GA6275	Gastric (ADC)

Target	Supplier #	Diln	Mw (kDa)	Species	Gel type	Gels probed	Comment
pKAP1 (S824)	Bethyl A300 767A	1:1000	110	Rabbit	BT	7a	
γH2AX (S139)	CST2577	1:1000	15	Rabbit	BT	6b	
pATM (S1981)	Abcam Ab81292	1:1000	360	Rabbit	TA	2	
Total ATM	Abcam ab78	1:1000	360	Mouse	TA	1	
Vinculin	Sigma V9131	1:10,000	116	Mouse	BOTH	all	Loading control for high mw- pico

Total ATM gel1

Model	5163	4248	5462	3464	6012	6307	2206	5862	4417	531	342				IR
Mouse	CR2506	CR3424	OV2029	BN2276	LU6473	GA2254	GA6275	PA1221	PA3023	LI6622	CR3280				NCI H460

NCI H460 irradiated cell lysate as +ve control for ATM

Total ATM

350kDa

Vinculin 117kDa

loading control

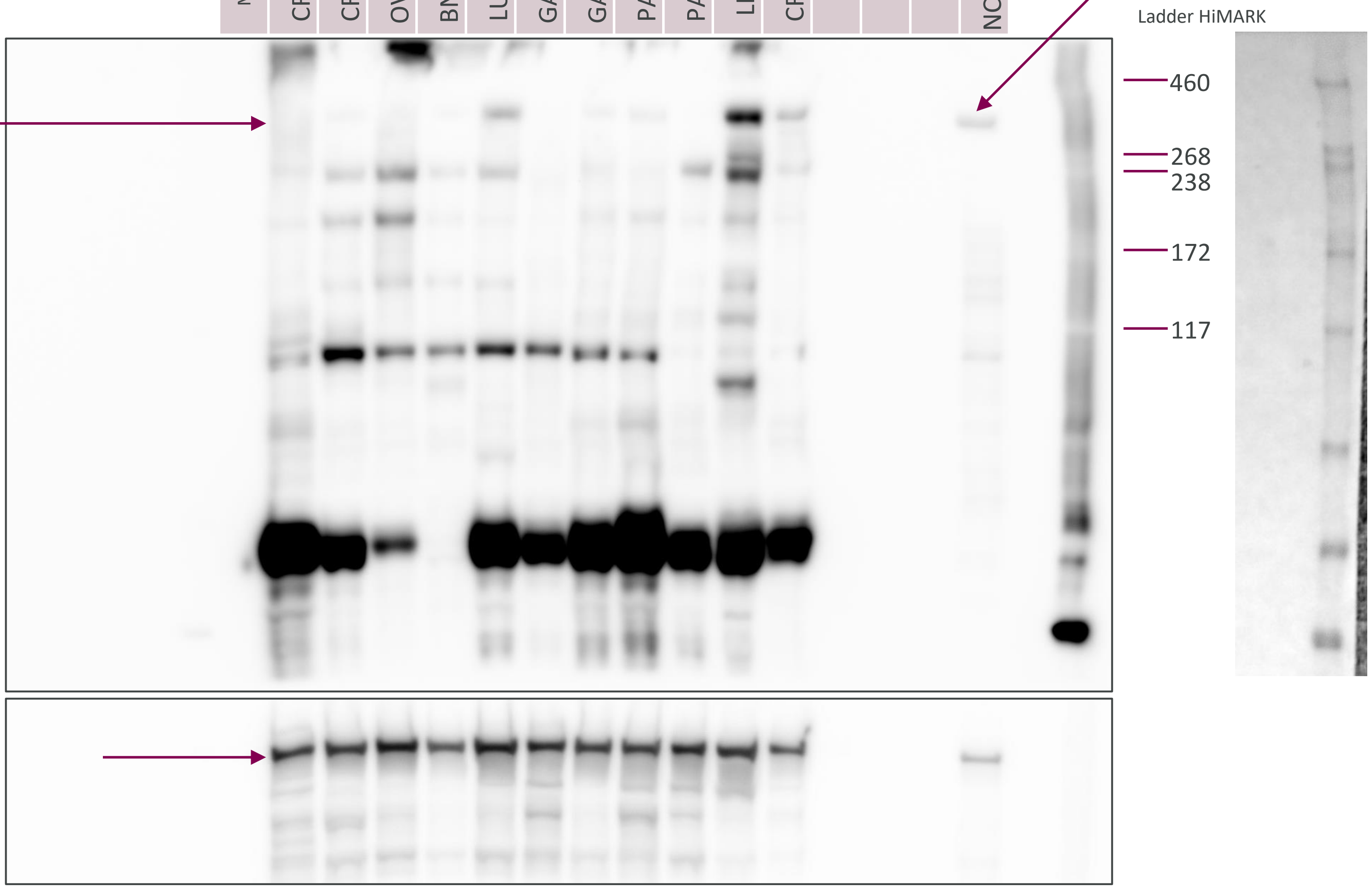

Light image of marker taken from same membrane (inverted)

pATM S1981

Model	5163	4248	5462	3464	6012	6307	2206	5862	4417	531	342				IR
Mouse	CR2506	CR3424	OV2029	BN2276	LU6473	GA2254	GA6275	PA1221	PA3023	LI6622	CR3280				NCI H460

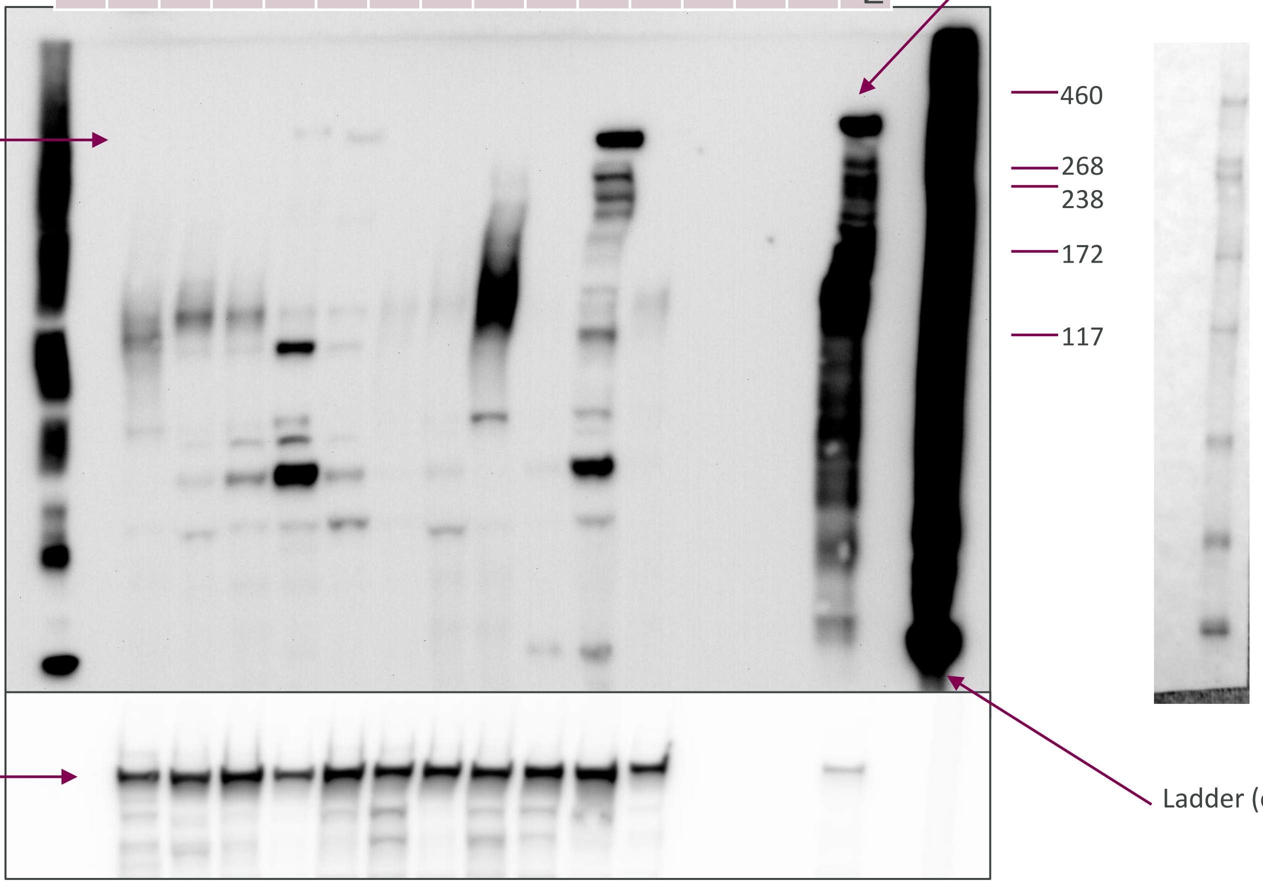

pKAP1 S824 gel 7A

Model	5163	4248	5462	3464	6012	6307	2206	5862	4417	531	342				IR
Mouse	CR2506	CR3424	OV2029	BN2276	LU6473	GA2254	GA6275	PA1221	PA3023	LI6622	CR3280				NCI H460

Ladder Magic MARK

Ladder HiMARK (overexposed)

pKAP1 S824

~110kDa

Vinculin 117kDa

loading control

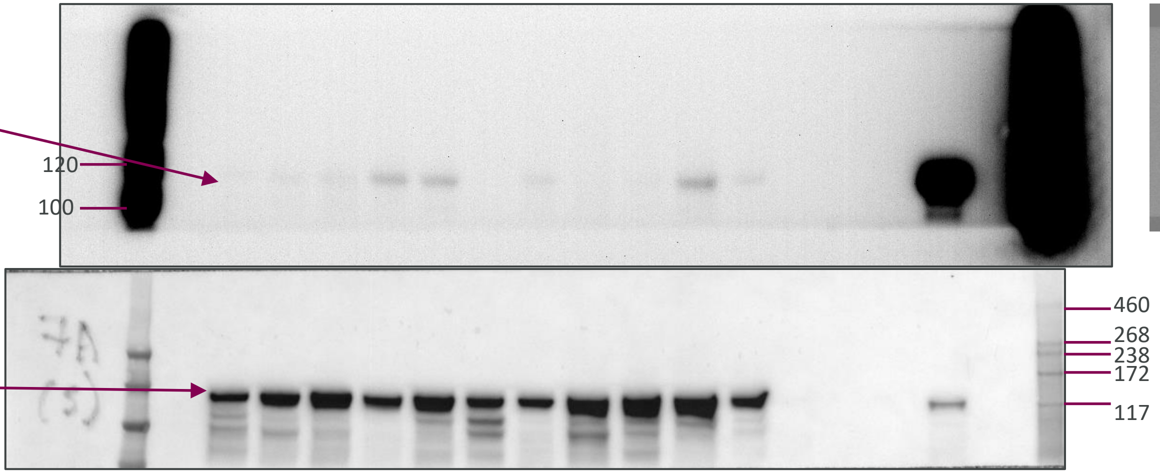

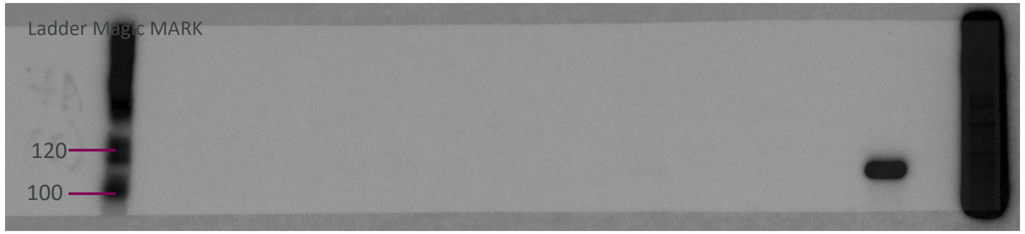

Short exposure to show ladder

Membranes cut into size appropriate fragments as also probed for other proteins

Vinculin probed after targets exposed

gH2AX S139 gel 6

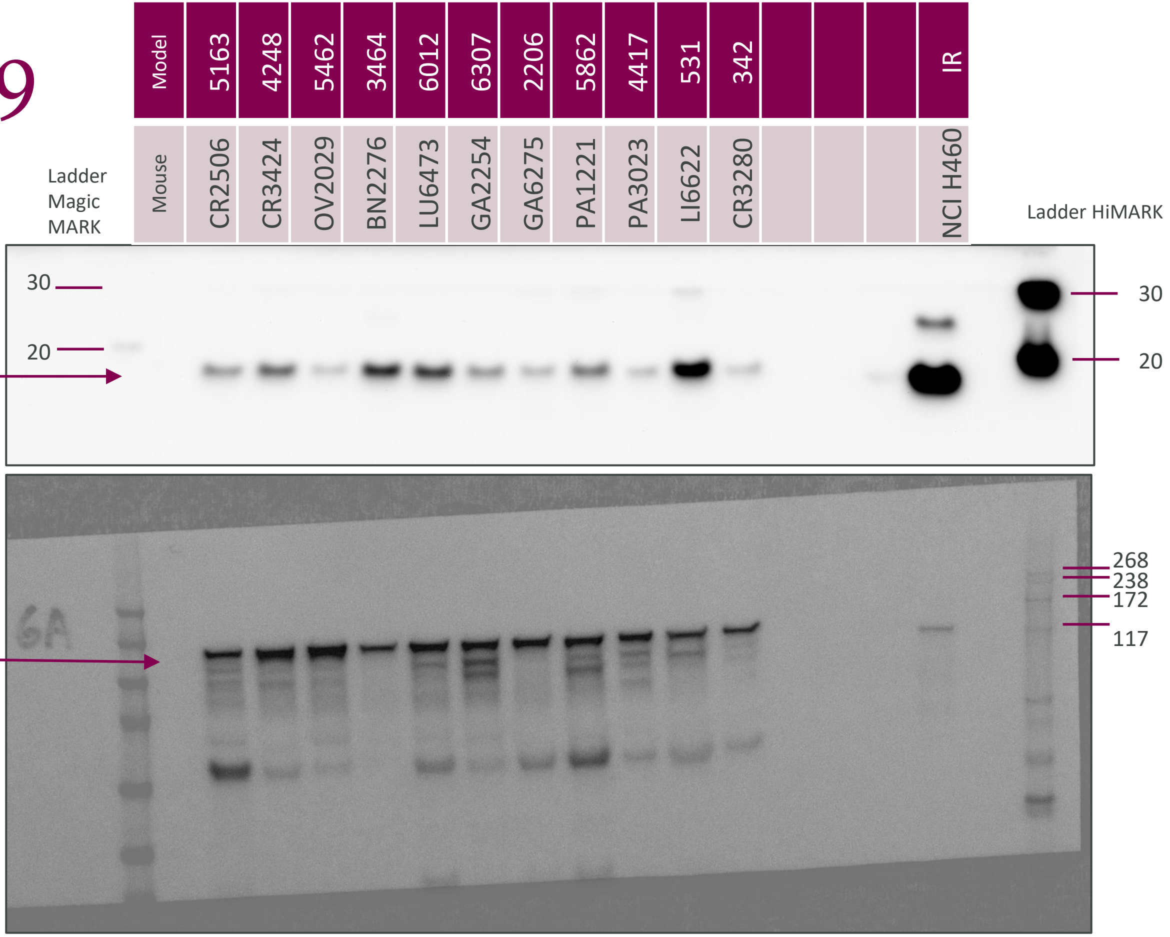

γH2AX S139

15kDa

Bottom fragment

Vinculin 117kDa

loading control

Top fragment

vinculin

Model	5163	4248	5462	3464	6012	6307	2206	5862	4417	531	342				IR
Mouse	CR2506	CR3424	OV2029	BN2276	LU6473	GA2254	GA6275	PA1221	PA3023	LI6622	CR3280				NCI H460

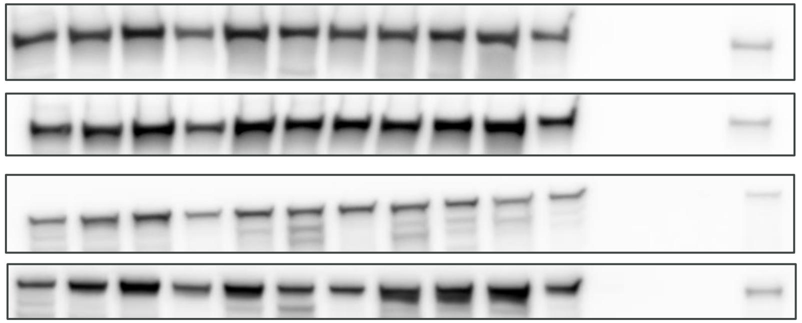

Gel1 vinc for total ATM

Gel2 vinc for pATM

Gel6 vinc for gH2AX

Gel7a vinc for pKAP1

Quantification

Model_ mouse	CR2506_5163	CR3424_4248	OV2029_5462	BN2276_3464	LU6473_6012	GA2254_6307	GA6275_2206	PA1221_5862	PA3023_4417	LI6622_531	CR3280_342
total ATM	36285072.0	6795426.0	20712196.0	1305648.0	76373984.0	0.0	10272352.0	16515102.0	0.0	138414368.0	54958508.0
pATM S1981	254204.0	308792.5	226876.0	788193.0	1315974.5	211291.0	241649.5	331589.0	74121.5	40658936.0	298389.0
gH2AX S139	11410284.0	17308324.0	5018477.5	26320416.0	24707254.0	11043535.0	6003770.5	16032612.0	4572134.5	45832120.0	5062465.5
pKAP1 S824	276905.0	379828.3	472593.0	1082456.8	1127321.3	52868.5	558158.5	273405.5	329459.0	1734198.3	433839.0

Model_ mouse	CR2506_5163	CR3424_4248	OV2029_5462	BN2276_3464	LU6473_6012	GA2254_6307	GA6275_2206	PA1221_5862	PA3023_4417	LI6622_531	CR3280_342
vinc for total ATM	35185624	31736636	38869732	19996966	38876772	26885282	24860932	26938072	27271308	31782330	23769140
vinc for pATM	36640784	37954128	47168800	25843744	46731996	35886052	36748200	38581552	41577024	49501168	33952532
vinc for gH2AX	18505724	21326808	25986698	10917126	22589734	24136678	16830570	20689764	16645518	17072006	12944868
vinc for pKAP1	44666944	53452584	73616488	40418196	71053000	49058312	36304628	72797800	74312720	86530000	47065448

Model_ mouse	CR2506_5163	CR3424_4248	OV2029_5462	BN2276_3464	LU6473_6012	GA2254_6307	GA6275_2206	PA1221_5862	PA3023_4417	LI6622_531	CR3280_342
total ATM/ vinc	1.03125	0.21412	0.53286	0.06529	1.96451	0.00000	0.41319	0.61308	0.00000	4.35507	2.31218
pATM / vinc	0.00694	0.00814	0.00481	0.03050	0.02816	0.00589	0.00658	0.00859	0.00178	0.82137	0.00879
gH2AX S139/ vinc	0.61658	0.81158	0.19312	2.41093	1.09374	0.45754	0.35672	0.77491	0.27468	2.68464	0.39108
pKAP1 S824/ vinc	0.00620	0.00711	0.00642	0.02678	0.01587	0.00108	0.01537	0.00376	0.00443	0.02004	0.00922

Confidentiality Notice

This file is private and may contain confidential and proprietary information. If you have received this file in error, please notify us and remove it from your system and note that you must not copy, distribute or take any action in reliance on it. Any unauthorized use or disclosure of the contents of this file is not permitted and may be unlawful. AstraZeneca PLC, 1 Francis Crick Avenue, Cambridge Biomedical Campus, Cambridge, CB2 0AA, UK, T: +44(0)203 749 5000, www.astrazeneca.com
